# Snapper: high-sensitive detection of methylation motifs based on Oxford Nanopore reads

**DOI:** 10.1093/bioinformatics/btad702

**Published:** 2023-11-20

**Authors:** Dmitry N Konanov, Vladislav V Babenko, Aleksandra M Belova, Arina G Madan, Daria I Boldyreva, Oksana E Glushenko, Ivan O Butenko, Dmitry E Fedorov, Alexander I Manolov, Danil V Krivonos, Vassilii N Lazarev, Vadim M Govorun, Elena N Ilina

**Affiliations:** Research Institute for Systems Biology and Medicine, Moscow 117246, Russia; Federal Research and Clinical Center of Physical-Chemical Medicine, Federal Medical Biological Agency, Moscow 119435, Russia; Federal Research and Clinical Center of Physical-Chemical Medicine, Federal Medical Biological Agency, Moscow 119435, Russia; Federal Research and Clinical Center of Physical-Chemical Medicine, Federal Medical Biological Agency, Moscow 119435, Russia; Department of Molecular and Translational Medicine, Moscow Institute of Physics and Technology, State University, Dolgoprudny 141700, Russia; Federal Research and Clinical Center of Physical-Chemical Medicine, Federal Medical Biological Agency, Moscow 119435, Russia; Research Institute for Systems Biology and Medicine, Moscow 117246, Russia; Research Institute for Systems Biology and Medicine, Moscow 117246, Russia; Research Institute for Systems Biology and Medicine, Moscow 117246, Russia; Research Institute for Systems Biology and Medicine, Moscow 117246, Russia; Research Institute for Systems Biology and Medicine, Moscow 117246, Russia; Department of Molecular and Translational Medicine, Moscow Institute of Physics and Technology, State University, Dolgoprudny 141700, Russia; Federal Research and Clinical Center of Physical-Chemical Medicine, Federal Medical Biological Agency, Moscow 119435, Russia; Research Institute for Systems Biology and Medicine, Moscow 117246, Russia; Research Institute for Systems Biology and Medicine, Moscow 117246, Russia

## Abstract

**Motivation:**

The Oxford Nanopore technology has a great potential for the analysis of methylated motifs in genomes, including whole-genome methylome profiling. However, we found that there are no methylation motifs detection algorithms, which would be sensitive enough and return deterministic results. Thus, the MEME suit does not extract all *Helicobacter pylori* methylation sites *de novo* even using the iterative approach implemented in the most up-to-date methylation analysis tool Nanodisco.

**Results:**

We present Snapper, a new highly sensitive approach, to extract methylation motif sequences based on a greedy motif selection algorithm. Snapper does not require manual control during the enrichment process and has enrichment sensitivity higher than MEME coupled with Tombo or Nanodisco instruments that was demonstrated on *H.pylori* strain J99 studied earlier by the PacBio technology and on four external datasets representing different bacterial species. We used Snapper to characterize the total methylome of a new *H.pylori* strain A45. At least four methylation sites that have not been described for *H.pylori* earlier were revealed. We experimentally confirmed the presence of a new CCAG-specific methyltransferase and inferred a gene encoding a new CCAAK-specific methyltransferase.

**Availability and implementation:**

Snapper is implemented using Python and is freely available as a pip package named “snapper-ont.” Also, Snapper and the demo dataset are available in Zenodo (10.5281/zenodo.10117651).

## 1 Introduction

Restriction-modification (R-M) systems are one of the essential mechanisms used by bacteria for protection from bacteriophage invasion. Generally, bacterial R-M systems are divided into four global groups (I, II, III, and IV) (Roberts *et al.* 2015) depending on the R-M complex structure. Despite their different architectures, all R-M systems share a common feature: the presence of site-specific DNA-binding domains that recognize specific short DNA sequence that should be methylated to prevent the restrictase activity.

The Oxford Nanopore technology (ONT) is a long-read sequencing technology based on measuring electric current across the nanopore (Jain *et al.* 2016). The ONT makes it possible not only to detect the four canonical nucleotide bases, but also to additionally catch any of their modified forms that can significantly change the sequencing signal (Liu *et al.* 2021). A number of tools aimed at accurate detection of methylated positions have already been developed (Stoiber *et al.* 2016, Liu *et al.* 2019, Ni *et al.* 2019, Tourancheau *et al.* 2021). However, these tools have certain limitations in inferring methylation site sequences, especially when dealing with low-represented DNA motifs. We faced it when we were analyzing our *Helicobacter pylori* data. In general, these tools provide accurate detection of methylated positions in a considered genome (Stoiber *et al.* 2016) or prediction of methylation type (6mA, 5mC, and 4mC) in a chosen site (Tourancheau *et al.* 2021) but we found that the process of precise identification of methylation motif sequences is either not sensitive enough or requires a lot of manual control.

Here, we present a novel tool called Snapper that performs high-sensitive identification of methylation motifs based on ONT sequencing data. The tool is mainly verified on *H.pylori* J99 sequencing data and compared with Tombo and Nanodisco, which are the most up-to-date instruments with similar functionality.

We chose *H.pylori* species as the object of interest in this study firstly because it is a unique organism that is shown to bring up to 30 different R-M systems in its genome (Gorrell and Kwok 2017) and has methyltransferases (MTases) capable of methylation adenine as well as cytosine (Gorrell and Kwok 2017). Secondly, it is a naturally competent organism (Hofreuter *et al.* 2001) that can be quite easily modified to confirm new inferred MTases. Third, the *H.pylori* J99 strain has been characterized earlier using the PacBio sequencing technology (Krebes *et al.* 2014) that allows us to directly compare our results with competing technology. To additionally validate the method, a few external datasets were analyzed to ensure that Snapper is capable of detecting methylation sites in different bacterial taxa.

To demonstrate the applicability of the developed tool, here, we use Snapper to characterize the methylome of a new *H.pylori* A45 strain. The method specificity is additionally demonstrated on A45-derived mutants disrupted in genes of MTases with known specificity.

## 2 Materials and methods

### 2.1 Bacterial strains and culture manipulation

The *H.pylori* A45 clinical isolate was obtained from a gastric mucosa biopsy sample of a patient with a gastric carcinoma (Momynaliev 2009). This isolate was used to obtain *hpy*, *hp91/92*, *hp1352*, *hp8*, and *hp944* derivatives defective in MTase genes (Supplementary Table S1). A targeted inactivation procedure was performed using the gene knockout method. The detailed description of *H.pylori* mutant strains construction is available in Supplementary Material S2.

*Escherichia coli* strain Top10 required for plasmid vectors assembly and production was cultivated at 37°C on solid Luria–Bertani medium or in liquid Luria–Bertani medium with aeration (150 rpm). The transformation procedure was carried out using the “heat shock” transformation protocol (https://international.neb.com/protocols/2012/05/21/transformation-protocol). Chloramphenicol (8 µg/ml, Panreac, Spain) and kanamycin (15 µg/ml, Sigma, USA) were added to the medium for selection.

*Helicobacter pylori* strains were cultivated for 20–48 h at 37°C under microaerophilic conditions on Columbian agar solid medium (Becton Dickinson, USA) supplemented with 10% donor horse serum (PAA Labs, Austria). Chloramphenicol (8 µg/ml) and kanamycin (15 µg/ml) were added for the selection and passage of resistant mutant strains. Amplicon cell transformation was carried out as described in Belova *et al.* (2018). Cells were cultivated for 24 h at 37°C under microaerophilic conditions on solid medium. The cell suspension (109 CFU) was moved on a new plate and cultivated for 4–5 h for undergrowth. Thereafter, sterile water suspension, containing 500 ng DNA fragments was applied dropwise onto the solid medium surface containing undergrowth cells. The dishes were then left agar side up in the CO_2_ incubator for 17–20 h. The next day, cells were subsequently transferred to a selective medium containing corresponding antibiotics. Clones found on plates after 4–5 days were verified to be *H.pylori* and to contain the specific insertions by PCR using primer sets listed in Supplementary Table S2. For any consequent assay all *H.pylori* strains were harvested from the plates after 2 days growth. Cells were washed with HBSS buffer, pH 7.5 (Hank’s Balanced Salt Solution, Thermo Fisher, USA) and precipitated by centrifugation at 3000 × *g* for 10 min for genomic DNA preparation as described earlier.

### 2.2 DNA manipulation

Vectors and mutant strains used in this study are listed in Supplementary Table S1. All standard methods of DNA manipulation, such as plasmid isolation by alkaline lysis, restriction endonuclease digestion, and ligation, were performed according to the protocols of Sambrook *et al.* (1989). *Helicobacter pylori* genomic DNA was prepared using the diaGene kit for DNA extraction from cell cultures (diaGene, Russia). Single DNA fragments or PCR amplification products for cloning or sequencing purposes were purified from agarose gels using Cleanup Standard Kit (Evrogen, Russia). DNA restriction enzymes were obtained from Fermentas (Thermo Fisher Scientific, USA) and were used according to the directions of the manufacturers.

### 2.3 Sequencing

#### 2.3.1 Whole-genome amplification

The Qiagen REPLI-g Single Cell Kit was used to perform whole-genome amplification to exclude epigenetically modified bases. The method produced micrograms of DNA from 10 ng of input genomic DNA, following the manufacturer’s guidelines and 8 h of amplification time at 30°C followed by deactivation at 65°C for 3 min.

#### 2.3.2 Oxford Nanopore

The DNA was isolated by using the Wizard DNA extraction kit (Promega Corporation, USA) and size selected with optimized solid phase reversible immobilization (SPRI) beads. The DNA concentration and quality were determined on a Qubit 4 Fluorometer and Nanodrop ND-1000 (Thermo Fisher Scientific). The long reads were generated with MinION sequencing (Oxford Nanopore Technologies, UK). The sequencing libraries were prepared using the ligation sequencing kit SQK-LSK109, native barcoding expansion kit EXP-NBD104 and run in a FLOMIN106 flow cell. Reads were basecalled using Guppy v3.6.1. using default parameters.

#### 2.3.3 Illumina

A NEBNext Ultra DNA library prep kit (New England Biolabs, USA) was used to prepare fragment libraries for genome sequencing. Sequencing was performed on the HiSeq 2500 System (Illumina, USA) HiSeq Rapid SBS Kit V2 using a 2 × 250 bp run configuration.

*De novo* assembly was performed by hybrid assembler Unicycler (v0.4.8) (Wick *et al.* 2017) using default parameters. Identification of the protein-coding sequences and primary annotation were performed using PROKKA v1.14.6 (Seemann 2014).

### 2.4 Methylation analysis

#### 2.4.1 Tombo

Raw signals from the fast5 files obtained for both native and WGS samples were mapped to corresponding reference genome positions using “tombo resquiggle” command (Stoiber *et al.* 2016). Next, modified positions were extracted using the “model sample compare” tombo mode. The resulting positions were used to construct all sequences that are likely to bring a methylation base, with the sequence length chosen to be 12, 18, and 22. These nucleotide contexts were processed by MEME (Bailey *et al.* 2015) with algorithm parameters recommended in the Tombo documentation and with customized parameters (-mod, -objfun, and -ng) in order to find the most sensitive approach. The motifs that were significantly over-represented in the selected contexts were considered as methylation sites.

Tombo was used only to analyze *H.pylori* J99 and four external datasets and as a result was excluded from the analysis of *H.pylori* A45 since its sensitivity turned out to be not enough for the analysis of highly methylated genomes.

#### 2.4.2 Nanodisco

For each strain, both sample and control fast5 files and the assembly file were processed according to the standard Nanodisco (v.1.0.3) pipeline described in the documentation (https://nanodisco.readthedocs.io/en/latest/overview.html). While running motif extraction stage (“nanodisco motif”), we manually controlled each motif inference iteration observing the pdf files generated by the tool since the automated mode turned out to be prone to occasional motif collisions. For external datasets, we used the motifs discovered by the authors *de novo* (listed in Supplementary Table S2).

#### 2.4.3 Snapper

**Figure 1.**
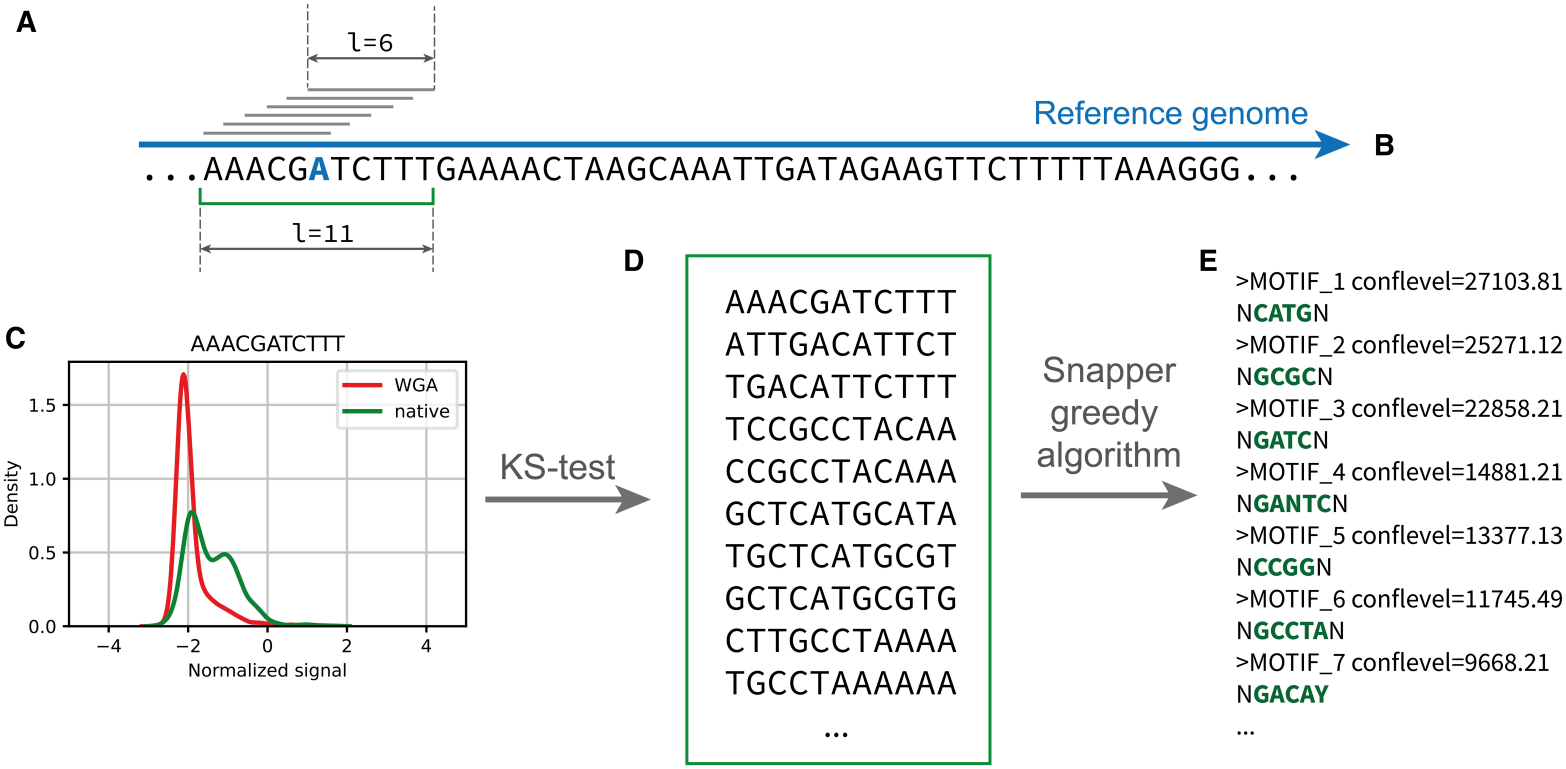
The principal scheme of the Snapper pipeline. In the first stage, for each *k*-mer in the reference genome (A and B), the algorithm collects normalized signal levels for this *k*-mer from multi-fast5 files for both native and WGA samples (here, *k* is chosen to be 11). Thereafter, for each *k*-mer presented in the genome, the algorithm directly compares the collected signal distributions (C) using the Kolmogorov–Smirnov test in order to select *k*-mers that most likely contain a modified base. The result of the first stage is an exhaustive set of all potentially modified *k*-mers (D). Next, the greedy motif enrichment algorithm implemented in Snapper iteratively extracts potential methylation motifs and calculates corresponding motif confidence levels (E). By default, motifs with confidence level >5000 are considered as significant but the authors recommend manually verifying extracted motifs with confidence levels lower than 3000 especially while the observed signal shift is rather weak. The main recommendations on how to interpret the Snapper results are available in the user guide (https://snapper-tutorial.readthedocs.io/en/latest/usercases.html#main-points)

Single-fast5 files obtained with “tombo resquiggle” were transformed to the multifast5 format to provide faster access to the sequencing raw signal data. These multifast5 files were processed using the python h5py library in order to collect signals for all *k*-mers presented in the considered genome for each strand independently. *k* was chosen to be 11 or higher (15 by default) in order to guarantee coverage of all 6-mers that cover an individual base (Fig. 1A). Thus, at first the algorithm generates a hash-table, where all presented *k*-mers are used as keys and corresponding normalized signal levels as values.

Next, for each *k*-mer in the hash-table, the Kolmogorov–Smirnov test is performed to compare signal level distributions between native and WGA samples (or, more generally, between any two samples passed as the input). Before the testing, the algorithm performs an artificial balancing of the sample sizes by random sampling (without replacement) of *N* values from each considered signal distribution to ensure the uniformity of the *k*-mers coverage (*N* was chosen to be 200). This balancing procedure is applied to make possible direct comparison of statistics values between *k*-mers, which are differently presented in the reference genome and to use the same statistics thresholds to infer modified positions along the genome. As a result, the algorithm returns the list of *k*-mers that are most likely to contain a modified base. In the further text, this list will be named *seq_set*. Simultaneously, the algorithm collects signal levels for long *k*-mers (with length of 29 by default). These long *k*-mers are used by the algorithm for additional motif refinement and enrichment of long motifs (mostly methylated by Type I R-M systems).

The next step differs from the classical motif enrichment approaches like the tools included in the MEME Suit. We have implemented a highly sensitive greedy algorithm that is aimed to generate the minimal set of short supermotifs of length 4–8 that can explain most part of sequences in the *seq_set*, under the assumption that all sequences in the *seq_set* contain at least one modified base. The principal idea of the algorithm is iterative search for the most over-represented motif in the *seq_set* in comparison with a reference genome using the χ^2^ statistics, extraction it as a potential modification motif, following procedure of motif adjustment, which additionally ensures the correctness and completeness of the extracted motif, and filtering the *seq_set* list by removing all sequences that contain the extracted motif variant. After the *seq_set* filtering, the search and extraction procedure repeats. The termination of the algorithm work is determined by the input parameters *confidence* and *max_motifs*, where the *confidence* parameter means the minimal value of χ^2^ test statistic (the default value is 5000 to provide high sensitivity), and the *max_motifs* parameter means the maximum desired number of extracted modification motifs (the default value is 20). As a result, the algorithm returns a sorted list of all potential motifs with corresponding confidence levels (we observed that typical confidence values for known methylation motifs are higher than 3000). A more formal explanation of the motif enrichment algorithm is available in Supplementary Material S3.

### 2.5 External data collection

Raw FAST5-files for *Thermacetogenium phaeum* DSM 12270, *Neisseria gonorrhoeae* FA 1090, *Methanospirillum hungatei* JF-1, and *Clostridium perfringens* ATCC 13124 were downloaded from SRP219538.

## 3 Results

### 3.1 Software description

We have developed a novel high-sensitive algorithm for methylation motifs detecting based on Oxford Nanopore sequencing data. The algorithm has been implemented as a command-line tool called Snapper and is available as a pip package to install. To perform non-target methylome analysis, the tool requires native and control (obtained by WGA) DNA samples being sequenced and resquiggled using Tombo. Snapper uses a *k*-mer approach, with *k* chosen to be at least 11 to guarantee coverage of all 6-mers that cover one particular base (Fig. 1A) under the assumption that, in general, ∼6 bases are located in the nanopore simultaneously (Zhang *et al.* 2021). By default, the *k* size is set to be 15 but can be tuned by the user. The raw signals data are extracted from the input fast5-files until the required minimal mean genome coverage depth is reached. The default value of the minimal coverage depth parameter was set to be 40×. This value was chosen since it showed a satisfactory convergence of the results on the external datasets, which will be described in detail below.

The first stage of the Snapper pipeline is the extraction of nucleotide short *k*-mers (typical *k* = 11–15, must be odd), which are most likely to contain a modified base. The comparison of signal distributions **(**Fig. 1C) is performed using the Kolmogorov–Smirnov test statistics. Next, all the extracted *k*-mers (Fig. 1D) are merged by a greedy algorithm, which generates the minimal set of potential modification motifs which can explain the most part of the selected *k*-mers (Fig. 1E), under the assumption that all selected *k*-mers contain at least one modified base. Snapper uses negative background for enrichment, and χ^2^ statistics is used to estimate a confidence level for the extracted motifs.

We observed that the typical confidence level for known motifs is 3000 or greater. Actually, the lowest score for a methylation motif among all the datasets, we have analyzed was 2307 (GTGAC motif for *H.pylori* J99, will be described below). We decided to set 500 as the default threshold to ensure that the tool is sensitive enough even for detection of very low-represented methylation motifs. The authors understand that such a low-threshold value could result in false-positive inference, so in the cases when the confidence level is rather low (lower than 3000) the user is recommended to check the motif correctness using signal shift plots generated by the tool.

The detailed algorithm explanation is available in Section 2 and Supplementary Material S3. A short tool’s guideline is available on https://snapper-tutorial.readthedocs.io. All the source code is available on GitHub (https://github.com/DNKonanov/Snapper).

In order to verify the approach developed, firstly we performed the total methylome analysis of *H.pylori* J99 strain, which has been characterized earlier using competing long-read sequencing technology PacBio (Krebes *et al.* 2014). Additionally, we compared our method with instruments Tombo (Stoiber *et al.* 2016) and Nanodisco (Tourancheau *et al.* 2021), which are in our knowledge the most up-to-date tools capable of non-target methylation motifs profiling besides Snapper. To check the capability of Snapper to deal with species different from *H.pylori*, a few external datasets representing different bacterial taxa were analyzed.

### 3.2 Total methylome analysis of *H.pylori* J99

The genome of *H.pylori* J99 (genotype cagA+/vacA s1m1) encodes about 20 R-M systems of Types II and III (Alm *et al.* 1999). At least 14 out of them have been shown to be active earlier using the PacBio long-reads sequencing (Krebes *et al.* 2014). We performed the full Snapper pipeline (the minimum coverage depth parameter was set 100×, other parameters were set by default) and discovered the same set of motifs except TCNNGA (Table 1, ONT+Snapper column). We should note that in Table 1, we demonstrate just the Snapper’s raw output. You can see more details in Supplementary Material S4. To verify the absence of TCNNGA motif, we manually observed a subset of genome contexts that contained this pattern and had a significant signal shift. We found that all such contexts contained other extracted motifs, such as GAGG, GATC, TCGA, and others. Thus, we concluded that despite the presence of TCNNGA in a number of methylated positions it is not an individual methylation motif in our J99 strain (more information about TCNNGA is available in Supplementary Material S5). In case of GTSAC and GWCAY motifs, we faced a certain ambiguity while motifs merging since GTCAC is a submotif for both these sites. Formally, such cases can be resolved only experimentally and it is the reason why we decided not to merge motifs automatically in the Snapper pipeline.

**Table 1.** Lists of J99 methylation motifs obtained using different methylation motif detection approaches.^a^

PacBio + MotifMaker	ONT + Snapper (raw output)	ONT + Tombo	ONT + Nanodisco	Total number in genome (±strand)
GAGG	GAGG		GAGG	2542/2462
GTSAC	GTCAC, GTGAC^b^			104/104
GATC	GATC		GATC	5499/5499
TCGA	TCGA			340/340
CCGG	CCGG		CCGG	1807/1807
ATTAAT	ATTAAT	ATTAAT	ATTAAT	426/426
CCNNGG	CCNNGG		CCNNGG	1193/1193
GTAC	GTAC		GTAC	182/182
CGWCG	CGACG, CGTCG^b^		CGWCG	264/264
GANTC	GANTC		GANTC	2756/2756
GCGC	GCGC	GCGC	GCGC	6104/6104
CATG	CATG	CATG	CATG	7567/7567
GCCTA	GCCTA	GCCTA	GCCTA	1532/1532
GWCAY	GACAY, GTCAT, GTCAC^b^	GACAC	GWCAY	2641/2464
GGWCNA	GGWCNA		GGWCHA	838/789
TCNNGA				1949/1949

a It should be noted here that the column entitled Snapper contains just the raw Snapper output excluding few false-positive motifs that were removed after manual results observation. The raw Snapper output and the verification process of this particular case are available in Supplementary Material S1. The PacBio results for *H.pylori* J99 were taken from the work of Krebes *et al.* (2014) directly (with few changes since the authors considered not the wild J99 strain but the J99-3 mutant). For Tombo, the best results that we managed to obtain with MEME are shown. For Nanodisco, the manually curated results are shown since the results generated in the automated mode were drastically less accurate.

b These motifs were extracted as different because the algorithm is designed to be quite cautious in motifs merging to prevent occasional motif collisions.

Additionally, we analyzed *H.pylori* J99 ONT reads using Tombo and Nanodisco tools, which are mainly designed at methylated position profiling but have their own motif enrichment functionality as well. Tombo uses the default MEME motif enrichment algorithm (Bailey *et al.* 2015) to extract motifs, so it has demonstrated rather low sensitivity and has inferred only five motifs highly represented in the genome (Table 1, ONT+Tombo column). Nanodisco also performs motif enrichment with MEME but uses an iterative approach that allows to detect more methylation sites but requires manual control on each motif extraction iteration because as we found in the fully automated mode Nanodisco is strongly prone to excessive motif collisions and even the most-represented motifs, such as CATG and GCGC, are not correctly discovered automatically and merged to GCRYG. Manually curated Nanodisco results are quite close to the automated Snapper output and the PacBio results except GTSAC and TCGA motifs (Table 1, ONT+Nanodisco column). Both these motifs are rather rare in the J99 genome, so the MEME algorithm did not identify them being enriched even using an iterative approach since Nanodisco uses the default MEME objective function without negative background (according to the Nanodisco source code). In the reference article, the authors also emphasize that GTAC has not been discovered in *de novo* mode due to the same reason. In Snapper, a set of all possible *k*-mers presented in the considered genome is used as a negative background, which simplifies the identification of rare motifs.

### 3.3 External FAST5 datasets analysis

Actually, the only dataset, which was used during the development of the core Snapper logic was *H.pylori* J99. To ensure that Snapper is not “overfitted” on *H.pylori* data, we downloaded external pair datasets containing WGA and native reads in FAST5-format for four different bacteria with highest number of different methylation motifs available in SRP219538 (Tourancheau *et al.* 2021): *T.phaeum* DSM 12270 (8 motifs), *N.gonorrhoeae* FA 1090 (8 motifs), *M.hungatei* JF-1 (6 motifs), and *C.perfringens* ATCC 13124 (6 motifs). *Helicobacter pylori* JP26 was not downloaded since here, we intentionally checked how the method works with non-*H.pylori* data. The genome coverage depth for each sample is listed in Table 2.

**Table 2.** The genome coverage depth, used in the methylome analysis of the four bacteria from external datasets.

Dataset	Native DNA coverage depth used	WGA DNA coverage depth used
*Thermacetogenium phaeum* DSM 12270	31×^a^	43×
*Neisseria gonorrhoeae* FA 1090	44×	41×
*Methanospirillum hungatei* JF-1	43×	41×
*Clostridium perfringens* ATCC 13124	42×	41×

a All available reads were used.

FAST5-files were resquiggled using Tombo and processed with Snapper with the default parameters. The results were almost identical to the reference REBASE motifs described for these strains except one long motif CACNNNNNRTAAA, which was inferred by Snapper as CACNNNNNATAAA (Supplementary Table S5). We manually checked the motif correctness and did not find significant signal shift for CACNNNNNGTAAA variant.

Few inaccuracies in motif identification were caused by motif collisions. Thus, in *M.hungatei* JF-1, which actually has AGCT and GCYYGAT motifs, Snapper identified AGCT, GCCTGAT, GCCCGAT, BGCTCGAT, and BGCTTGAC motif sequences. Here, AGCT had been extracted earlier that led to omitting AGCTCGAT and AGCTTGAC variants, and GCYYGAT motif was extracted formally incomplete. Generally, the algorithm cannot resolve such collisions without additional information and does not try to merge them to prevent results ambiguity. The same problem appeared in *N.gonorrhoeae* FA 1090, where RGCGCY motif was extracted incompletely since its GGCGCC subvariant intersected with GGNNCC motif sequence extracted first, so the Snapper output included only GGNNCC, AGCGCC, AGCGCT, and GGCGCT sequences but not GGCGCC.

Interestingly, Snapper inferred a few methylation sites that were not mentioned in the reference article (Tourancheau *et al.* 2021). Thus, we observed significant signal shift and quite high confidence level for CCAG and TGGCCA motifs in *T.phaeum* DSM 12270. Both these motifs were independently approved using Nanodisco software in manual mode using the “motif refine” command (motif refinement plots are available in Supplementary Material S6). The WGGCCW motif not mentioned in the reference article was found in *C.perfringens* ATCC 13124.

Also, we analyzed the methylome of the four chosen bacteria with Tombo. Here, to detect methylated positions, all available data were used. We tried to use different parameters for both Tombo processing (the *k*-mer size, the number of extracted sequences) and MEME motif enrichment (the objective function, mode) but have managed to identify only two correct motifs for *T.phaeum* DSM 12270, and one correct motif for three other bacteria. The detected motifs are presented in Supplementary Table S5. Probably, the iterative approach similar to implemented in Nanodisco would provide better results but we intentionally tried to follow only the instructions from the Tombo’s documentation.

### 3.4 Total methylome analysis of *H.pylori* A45 and mutants

The *H.pylori* A45 (genotype cagA-/vacA s2m2) clinical isolate was obtained previously from a gastric mucosa biopsy sample from a patient with a gastric carcinoma (Momynaliev 2009). In addition, the derivatives of the A45 strain of *H.pylori*, defective in MTase genes M.HpyAI (*hpy* mutant), M.HpyAIII (*hp91/92* mutant), and M.HpyAIV (*hp1352* mutant) were obtained (Momynaliev 2009). As well as for *H.pylori* J99, here, we set the minimal genome coverage parameter as 100×. For the native sample the tool collected raw signals with the coverage depth of 101× but the control sample had the mean coverage of 61× due to lower sequencing depth.

The analysis of *H.pylori* A45 has revealed presence of 15 methylation sites belonging to R-M systems of types II and III: ATTAAT, GTNNAC, GGRGA, CCATC, CATG, CCAG, GCGC, GANTC, GATC, GGCC, GAAC, TGCA, TCGA, TCNGA, and TCNNGA (Table 3, wild-type column). Interestingly, opposite to the *H.pylori* J99 analysis, in this case, all the extracted motifs had quite high confidence levels >3000 (Supplementary Material S7). Despite that, we manually observed signal distributions for all suggested sites and their closest genome context to ensure that all motifs had been extracted correctly.

**Table 3.** Methylation motifs detected in the wild A45 and four mutants.^a^

Wild-type	Total number in genome (+/− strand)	*hpy*	*hp1352*	*hp91/92*	*hp944*
ATTAAT	441/441	ATTAAT	ATTAAT	ATTAAT	ATTAAT
GTNNAC	325/325	GTNNAC	GTNNAC	GTNNAC	GTNNAC
GGRGA	1768/1790	GGRGA	GGRGA	GGRGA	GGRGA
CCATC	1163/1115	CCATC	CCATC	CCATC	CCATC
CATG	7246/7246		CATG	CATG	CATG
CCAG	2173/2271	CCAG	CCAG	CCAG	
GCGC	5982/5982	GCGC	GCGC	GCGC	GCGC
GANTC	2625/2625	GANTC		GANTC	GANTC
GATC	5113/5113	GATC	GATC		GATC
GGCC	1534/1534	GGCC	GGCC	GGCC	GGCC
GAAC	2760/2700	GAAC	GAAC	GAAC	GAAC
TGCA	5770/5770	TGCA	TGCA	TGCA	TGCA
TCGA	290/290	TCGA	TCGA	TCGA	TCGA
TCNGA	1224/1224	TCNGA	TCNGA	TCNGA	TCNGA
TCNNGA	1926/1926	TCNNGA	TCNNGA	TCNNGA	TCNNGA
	3403/3267	CCAAK		CCAAK	

a Interestingly, in the *hpy* and *hp91/92* mutants an additional CCAAK methylation motif is observed.

To identify the MTases responsible for methylation of new sites CCAG, GGRGA, and GAAC not described earlier for *H.pylori*, which were presented in *H.pylori* A45 and absent in *H.pylori* J99, all candidate MTase genes were extracted from both genomes and used to construct a phylogenetic tree based on protein sequences similarity. Two proteins that were presented only in *H.pylori* A45 strain and did not have orthologous genes in *H.pylori* J99 were considered as potentially new MTases. Two new *H.pylori* A45 derived mutants (*hp8* and *hp944*) disrupted in corresponding genes hp0008 and hp0944 were obtained during this work. Their native DNA was analyzed using ONT and the sequencing data were processed using Snapper. The *hp8* mutant did not have any signal changes, but in the *hp944* mutant we observed a successful deactivation of CCAG-specific MTase (Table 3, *hp944* column). As we found later, the negative result for *hp8* was caused by a nonsense mutation in the MTase gene in the wild A45.

**Figure 2.**
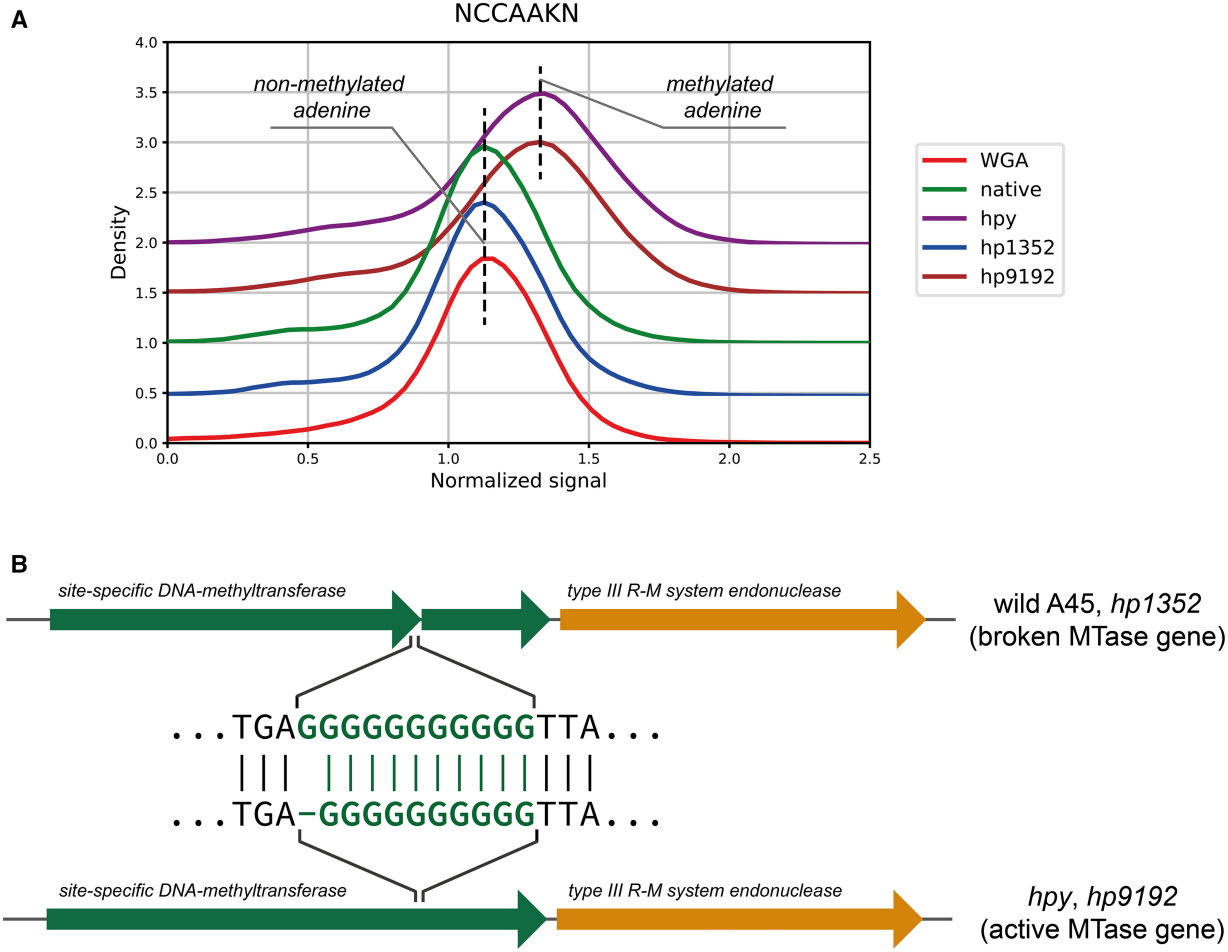
CCAAK-specific MTase inference. (A) Normalized signal distributions for the CCAAK methylation site. *hp91/92* and *hpy* mutants have a visible signal shift in comparison with the WGA sample while the wild A45 and the *hp1352* mutant have signal distributions identical to the WGA sample. (B) Wild A45 and the *hp1352* mutant have CCAAK-specific MTase gene broken because of a frame-shift insertion in a homopolymeric region in the gene. The reverse frame-shift mutation in the MTase gene restores the Type III R-M system activity in *hpy* and *hp91/92*

Here, to estimate the method specificity, in addition to the wild-type, we analyzed three mutants of *H.pylori* A45 disrupted in three different genes encoding MTases with known specificity (*hpy*, *hp1352*, and *hp91/92* mutants disrupted in the genes of MTases specific to CATG, GANTC, and GATC motifs, respectively). Here, we used as a control not the WGA sample but the native A45 to check out how the algorithm works with a small number of motifs that differ by their signal level. We expected the algorithm to extract only one motif for each mutant, but two mutants had an additional motif that seemed to be modified. Firstly, in all three mutants Snapper successfully detected the absence of methylation of the corresponding motifs (Table 3, *hpy*, *hp1352*, and *hp91/92* columns, and Supplementary Fig. S5). Secondly, the CCAAK site was detected as methylated in *hpy* and *hp91/92* mutants while the native sample, *hp1352*, and *hp944* had CCAAK signal distributions identical to the WGA sample (Fig. 2A).

To identify the MTase responsible for modification of CCAAK, we used non-target proteomics data that were obtained earlier for the wild A45 and *hpy*, *hp1352*, and *hp91/92* mutants (the procedure is described in Supplementary Material S14). Five proteins that were significantly over-represented (LFC≥5) in *hpy* and *hp91/92* mutants in comparison to the wild-type and *hp1352* were found and manually annotated using BLASTP (Supplementary Tables S11 and S12). Proteins encoded by hp0008 and hp0009 genes were annotated as two fragments of one MTase gene, and gene hp0010 was annotated as a Type III R-M system endonuclease, so, these three genes encode a full Type III R-M system but with a broken MTase gene. The appearance of the CCAAK methylation site in *hpy* and *hp91/92* was caused by a reverse frame-shift mutation in a homopolymeric region in hp0008 gene, which was confirmed by short Illumina reads (Fig. 2B and Supplementary Fig. S6).

Additionally, we checked the coverage depth required for extraction of two non-overlapped motifs (the *hpy* mutant, CATG and CCAAK motifs). The tool extracted these motifs correctly with mean coverage depth of 14× (∼20 000 reads per sample).

## 4 Discussion

Oxford Nanopore sequencing has a great potential for the analysis of epigenetic modifications of the four canonical bases. Unfortunately, the current software designed for methylation site detection possesses certain drawbacks. Thus, both Tombo and Nanodisco provide high-quality profiling of methylated positions in a genome but the main problem here is to accurately generate a set of potential motifs that could explain all methylated bases. Technically, it is a classical motif enrichment task, but classical approaches have proven to be insufficiently sensitive for analyzing highly methylated genomes, such as the *H.pylori* genome. Thus, we found that the most widely used tool suit MEME is capable of detecting only highly represented motifs. An iterative approach implemented in Nanodisco is more sensitive but requires a lot of manual work while extracting motifs, which limits its applicability when more than few genomes are studied.

We have developed a new greedy algorithm that is aimed at high-sensitive motif enrichment. This algorithm has been implemented as the command-line tool Snapper. Here, we have shown that, firstly, as a fully automated pipeline, Snapper outperforms both Tombo and Nanodisco coupled with the MEME tool and, secondly, Snapper is capable of detecting methylation site sequences with sensitivity equivalent to the PacBio technology and is slightly more sensitive than Nanodisco in the manual mode. In contrast to Nanodisco, Snapper provides a fully automated motif extraction algorithm and requires manual results verification only during adjustment of motifs with a low confidence level.

We should note again that Snapper must not be considered as an alternative to MEME since MEME has been developed as a universal approach and can deal with any type of data, while Snapper uses special search heuristics aimed at finding methylation motifs specifically. These heuristics narrow the search space, and as a result, the algorithm can provide higher enrichment sensitivity.

Another feature we should note is an intended algorithm cautiousness in motifs merging to avoid occasional motif collisions. Thus, the *H.pylori J99* analysis has shown the presence of motifs GTCAT, GTCAC, GACAY, and GTGAC as methylated. Actually, these submotifs are explained by GWCAY and GTSAC methylation sites but GWCAY+GTGAC variant is formally possible as well. Such cases can be resolved only experimentally so we decided not to merge controversial motifs in the Snapper motif extraction algorithm. A related problem with motif sequences resolving appeared in the *M.hungatei* JF-1 analysis results, which actually has AGCT and GCYYGAT methylation sites but Snapper’s output included AGCT, GCCCGAT, GCCTGAT, BGCTCGAT, and BGCTTGAC (“B” means “not A”). Indeed, the last two motif subvariants were inferred after AGCT had been inferred. Therefore, the algorithm had already explained AGCTCGAT and AGCTTGAC subvariants by the presence of AGCT. Thus, although the algorithm is formally fully automated, some cases should be considered manually.

Since Snapper aims not at the methylated positions calling but methylation motif sequences, it is relatively less demanding on the input data compared with Nanodisco or Tombo. The genome coverage required for the analysis depends on the object of interest. Thus, while analyzing organisms that have very few different non-overlapped methylation sites, the method can work well even with a mean genome coverage of 15–20×. On the other hand, for organisms with higher number of different methylatinon sites Snapper requires higher sequencing coverage to more accurately resolve similar motif sequences. The authors suggest that, in most cases, coverage of 80–100× is absolutely enough for high-accurate methylation motifs detection. On the other hand, since Snapper is not aimed to detect modified positions and predict the modification type, the authors recommend using Nanodisco for these purposes after the target motif sequences have been identified.

Using Snapper, we fully characterized the methylome of a new *H.pylori* strain A45. In this strain, we found three methylation sites that have not been described earlier for *H.pylori* (GGRGA, GAAC, and CCAG) and managed to experimentally confirm the MTase specific to CCAG. In addition, during the experiment, we observed a frame-shift-based phase variation in the gene encoding a new MTase specific to CCAAK methylation site. Thus, we did not observe this site being methylated in the wild A45 culture but it appeared after the disruption in CATG-specific MTase or GATC-specific MTase. In both cases, it was caused by the same reverse frame-shift mutation in a homopolymeric region in the gene encoding corresponding MTase. Such a behavior is quite typical for *H.pylori* and is often used to regulate activity of particular MTase genes (Beaulaurier *et al.* 2015).

As an additional verification, we analyzed four external datasets representing different bacterial taxa. In all cases, Snapper discovered all methylation motif sequences that had been shown for these bacteria according to the REBASE database and few additional motifs, which has illustrated the versatility of the method. However, we observed few problems with motif sequences resolving (as the AGCT and GCYYGAT collision mentioned above), especially in highly methylated organisms, so we cannot declare that Snapper’s results can always be used as is and should be additionally curated by a user.

## 5 Conclusion

We have developed Snapper—a tool for high-sensitive methylation motifs identification. Snapper has been shown to be more sensitive than MEME coupled with the Tombo software. Snapper demonstrated slightly higher motif detection accuracy compared to Nanodisco and did not require extensive manual work during the motif extraction procedure, unlike Nanodisco. Snapper was tested in detail on data obtained in this study as well as on a few external datasets. Using Snapper, we characterized the methylation motifs in a new *H.pylori* A45 strain and discovered four motif sequences that have not been described for *H.pylori* earlier. We managed to experimentally confirm a gene encoding a new CCAG-specific MTase and inferred a gene encoding CCAAK-specific MTase.

## Supplementary Material

btad702_Supplementary_Data

## Data Availability

All the source code is available on Github (https://github.com/DNKonanov/Snapper). Fastq files are available in SRA (NCBI Bioproject accession number PRJNA175046). The mass spectrometry proteomics data have been deposited to the ProteomeXchange Consortium via the PRIDE (Perez-Riverol *et al.* 2019) partner repository with the dataset identifier PXD038899 (access for the reviewers: login reviewer pxd038899@ebi.ac.uk, password—xOuk64qK). All genome assemblies obtained during this study are available in GenBank (*Helicobacter pylori* A45 wild-type: GCA 000333835.2, *hpy*: GCA 013122115.1, *hp1352*: GCA 013122055.1, *hp91/92*: GCA 013122035.1). External fast5-files used in this study were downloaded from SRP219538. Additionally, we provide a small fast5 example dataset in order to demonstrate the tool capabilities. The demo-dataset includes 10 + 10 preprocessed multi-fast5 files containing 40 000 long reads for the hpy mutant and 40 000 reads for the wild A45 as a control (http://download.ripcm.com/snapper_test). Both Snapper tool and the demo dataset are available in Zenodo (10.5281/zenodo.10117651).
