# Correction to: Snapper: high-sensitive detection of methylation motifs based on Oxford Nanopore reads

**DOI:** 10.1093/bioinformatics/btad770

**Published:** 2023-12-25

This is a correction to: Dmitry N Konanov, Vladislav V Babenko, Aleksandra M Belova, Arina G Madan, Daria I Boldyreva, Oksana E Glushenko, Ivan O Butenko, Dmitry E Fedorov, Alexander I Manolov, Danil V Krivonos, Vassilii N Lazarev, Vadim M Govorun, Elena N Ilina, Snapper: high-sensitive detection of methylation motifs based on Oxford Nanopore reads, *Bioinformatics*, Volume 39, Issue 11, November 2023, btad702, https://doi.org/10.1093/bioinformatics/btad702.

In the originally published version of this manuscript, there was a typographic error in a link within the ‘Data availability’ section, which read ‘http://download.ripcm.com/snappertest’. This has been corrected to ‘http://download.ripcm.com/snapper_test’.

