# Supplementary material for: Snapper: high-sensitive detection of methylation motifs based on Oxford Nanopore reads
Source: Bioinformatics. 2023 Nov 20;39(11):btad702. doi: 10.1093/bioinformatics/btad702 (PMC10689673; doi:10.1093/bioinformatics/btad702)
Supplement: btad702_Supplementary_Data [file btad702_supplementary_data.pdf]

1. Supplementary methods

As we have only one promoter region P_{Cm} from *catGC* cassette, successfully expressing in *H. pylori* cells, we used this promoter in both cases: as a part of chloramphenicol resistant amplicon and fused with promoterless kanamycin resistance gene for kanamycin amplicon. The *catGC* cassette and chloramphenicol resistance gene promoter region P_{Cm} were amplified from the plasmid pHel2 [1] using ACm-F/ACm-R and ACmp-F/ACmp-R primer set, respectively. Promoterless kanamycin resistance gene were amplified from the plasmid pEGFP-N1 (https://www.addgene.org/vector-database/2491/) using AKan-F/AKan-R primer set (Supplementary Table 7). All inner primers were constructed in an overlapping manner for latter two-step PCR amplification of full-length amplicons. (Supplementary Figure 5).

Two-step PCR amplification was realized as follows. On the first step two *H. pylori* flanking fragments and resistance gene fragment in equal concentrations 10 ng were incubated without primers in a final volume of 45 µL containing 1x Tersus plus buffer, 100 µmmol/L deoxynucleoside triphosphate, 0.4 U of Tersus polymerase (Evrogen, Russia) in thermocycler T100 (BioRad, USA) for 8-10 cycles for overlapping fragment parts could «stick» to each other. At the second stage, the appropriate primers, 20 µmmol/L of each primer, were added and mixtures were amplified for 25 cycles (Supplementary Figure 6). In both cases two-step PCR amplification resulted in two full-length fragments 1671 bp for *hp0008* and 1567 bp for *hp0944* gene disruption, respectively.

The amplicon was purified using Cleanup Standard Kit (Evrogen, Russia), cloned into the pCR2.1 vector system using The Original TA Cloning Kit (Invitrogen, USA) according to the manufacturer's recommendations and transformed into *E. coli* Top10 strain. Cloning was verified by PCR with the M13F/R vector-based primer set (Supplementary Table 7). The plasmids pCR2.1-8, pCR2.1-9 containing the expected amplicon (Supplementary Table 8) were purified using the QIAprep® Spin Miniprep Kit (Qiagen, Germany) and sequenced. Plasmids with verified amplicon nucleotide sequence were digested with restriction enzymes using XbaI/KpnI sites for *hp0008* gene disruption amplicon and KpnI/NotI sites for *hp0944* gene disruption amplicon. Amplicons were purified from agarose gel in appropriate concentrations and used to transform *H. pylori* A45 cells as described earlier.

Supplementary Table 1. Plasmids and strains used in this study.

**Plasmid/Strain**	**Description**	**References**
PLASMIDS		
pGEM-HpyIM-к	pGEM-T-Easy vector system containing full-lenth amplicon for *HpyAI* gene disruption in *H. pylori* A45 strain	[2]
pGEM-HpyIIIM	pSK-Kn vector system containing full-lenth amplicon for *hp0091/hp0092* genes disruption in *H. pylori* A45 strain	[2]

pSK-HpylVM	pGEM-T-Easy vector system containing full-lenth amplicon for *hp1352* gene disruption in *H. pylori* A45 strain	[2]
pCR2.1-8	pCR2.1 vector system containing full-lenth amplicon for *hp0008* gene disruption in *H. pylori* A45 strain.	This study
pCR2.1-9	pCR2.1 vector system containing full-lenth amplicon for *hp0944* gene disruption in *H. pylori* A45 strain.	This study
H. PYLORI STRAINS		
A45	WT	[2]
hpy	A45 mutant strain with disrupted in the HpyAI gene by homologous recombination	[2]
hp91/92	A45 mutant strain with disrupted in the *hp0091/hp0092* genes by homologous recombination	[2]
hp1352	A45 mutant strain with disrupted in the *hp1352* gene by homologous recombination	[2]
A458 (*hp8*)	A45 mutant strain with disrupted in the *hp0008* gene by homologous recombination	This study
A459 (*hp944*)	A45 mutant strain with disrupted in the *hp0944* gene by homologous recombination	This study

Supplementary Table 2. Oligonucleotides used in this study.

**Primer set**	**Sequence**	**Product**
Hs9441-F<br>Hs9441-R	AATCCACACCCTTTGGATAACT<br>GGGGCGTAACTAATGGAGCGTGGATTAGCA	hp0944 upper region<br>353 bp
ACm-F<br>ACm-R	ACGCTCCATTAGTTACGCCCCGCCCTGCCA<br>GTGAGCTTAAACCGCCATATTGTGTTGAAACAC	chloramphenicol resistance cassette
Hs9442-F<br>Hs9442-R	ACAATATGGCGGTTTAAGCTCACTTGTCCGTC<br>GGGAGATAGAAGCATAGAAGTT	hp0944 lower region<br>456 bp

Hs82-F<br>Hs82-R	TCACGCCGTCTTGTTTGAGC<br>TTTGAGAACTTAAACGAATCCACTAGCAAG	hp0008 upper region<br>463 bp
AKan-F<br>AKan-R	GTTTAAGTTCTCAAAATCAGAAGAACTCGT<br>AGCTAAAATGATTGAACAAGATGGATTGCA	promoterless kanamycin resistance gene
ACmp-F<br>ACmp-R	TCAATCATTTTAGCTTCCTTAGCTCCTGAA<br>CTGGATACACGCCATATTGTGTTGAAACA	chloramphenicol promoter region
Hs81-F<br>Hs81-R	CAATATGGCGTGTATCCAGCGTGAGCTTATC<br>AGGCTTAGCGACTTTACACCA	hp0008 lower region<br>325 bp
23s-F<br>23s-R	GTAAGCCATAGAAAGTGATAGCCT<br>CTTAACCTTGCCAGATACCACA	primers for verification (complimentary to 23s RNA H. pylori gene)
Cms-F<br>Cms-R	ATCCCATATCACCAGCTCAC<br>GGAATTCCGTATGGCAATG	primers for verification (complimentary to chloramphenicol resistance gene)
M13f<br>M13r	TGTAAAACGACGGCCAGT<br>CAGGAAACAGCTATGAC	primers for verification (complimentary to pCR2.1 vector)

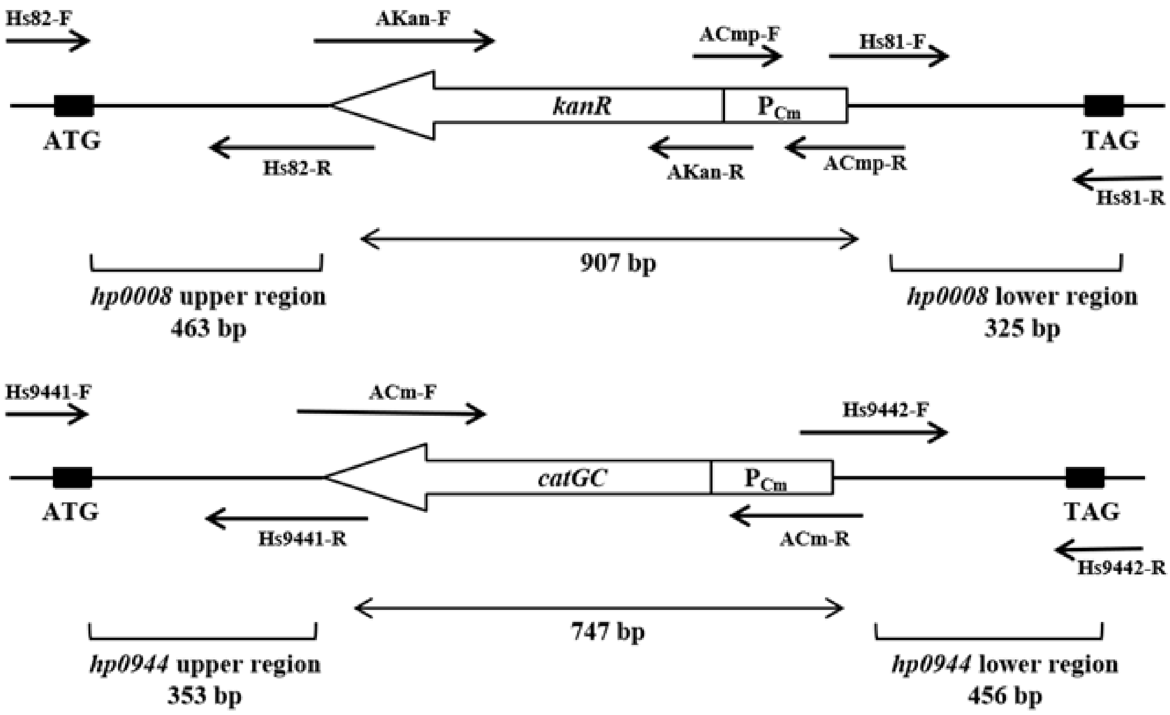

Supplementary Figure 1. Schematic map of the primer location and amplicons structure used for hp0008 and hp0944 gene disruption in *H. pylori* A45 strain.

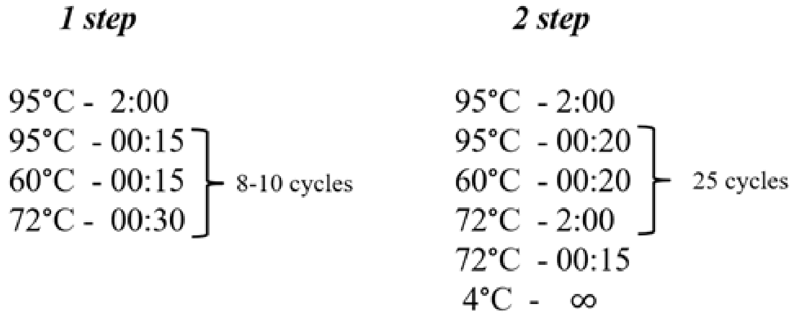

Supplementary Figure 2. Two-step PCR conditions used for amplification of full-length fragments 1671 bp for *hp0008* and 1567 bp for *hp0944* gene disruption in *H. pylori* A45 strain, respectively .

2. *H. pylori* mutant strains construction. Plasmids used for gene inactivation restriction-modification systems

H. pylori mutant strains *hpy*, *hp91/92*, and *hp1352* were obtained earlier [11]. A cassette containing kanamycin resistance gene (aphA-3) as a selectable marker was inserted into the target region of the genome. For a detailed description of the plasmids used to inactivate the genes involved in restriction-modification systems, see Supplementary Materials Table 9 (3). Validation of the correct insertion of the cassette into the H. pylori genome was confirmed by PCR-analysis and sequencing of the obtained strains. Target inactivation - genes encoding methyltransferases was confirmed by restriction analysis of genomic DNA of the obtained strains using methyl-sensitive restriction

endonucleases: Mbol (GATC), Hin1ll (CATG), Hinfl (GANTC). *H. pylori* mutant strains *hp8*, *hp944* were obtained during this work by homologous recombination. For construction *hp944* mutant strain (A45 strain disrupted in the hp0944 gene) the amplicon, consisting of chloramphenicol resistance catGC cassette flanked by two fragments of the hp0944 of 363 bp and 456 bp, was obtained by two-step PCR amplification. For construction hp8 mutant strain (A45 strain disrupted in the hp0008 gene) the amplicon, consisting of kanamycin resistance gene under chloramphenicol promoter region flanked by two fragments of the hp0008 gene of 325 bp and 463 bp, was obtained by two-step PCR amplification. All *H. pylori* flanking regions were amplified from genomic DNA of A45 strain using Tersus polymerase kit (Evrogen, Russia) according to the directions of the manufacturers. All corresponding primer sets listed in Supplementary Table 7 (2). Schematic map of the primer location and amplicon structure used for hp0008 and hp0944 gene disruption in *H. pylori* A45 strain illustrated by Supplementary Figure 5 (1) in Supplementary Materials.

Plasmid Construction pGEM-HpyIM-к (*hpy*)

Two fragments of the gene encoding HpyAI methyltransferase of *H. pylori* strain A45 labeled IMl and IM2 were amplified using primers 1IMfw, 1IMrev, 2IMfvv, 2IMrev (Supplementary Table 9). For the full-length gene assembly, the following reaction mixture was composed: 200 ng of each IM1 and IM2 fragments, 20 pM of each primers 1lmfw and 2Imrev, 2mM dNTPs, 1xPCR buffer, 4u of Taq polymerase. PCR was performed with the following amplification conditions: 94°C – 30 sec, 45°C – 1 min, 72°C – 1 min. 30 sec. A total of 35 amplification cycles were performed. The resulting fragment, after being treated with Xhol restriction enzyme, was cloned into the pGEM-T-Easy plasmid (Promega, USA) which resulted in formation of the plasmid pGEM-HpylM. The *aphA-3* kanamycin resistance gene was amplified from the pHel3 plasmid [1] using primers X-aph-fw and X-aph-rev, then the resulting fragment was digested with restriction enzyme Xhol and cloned into pGEM-HpylM plasmid treated with Xhol restriction enzyme, which resulted in formation of the plasmid pGEM-HpyIM-k.

Plasmid Construction pGEM-HpyIIIM (*hp91/92*)

Fragments of the *hp0091* and *hp0092* genes of the A45 *H.pylori* strain labeled 91 and 92 were amplified using primers 91fw, 91revKn, 92fw, 92revKn (Supplementary Table 9). The kanamycin resistance gene (aphA-3) was amplified from the pHel3 plasmid [1] using primers Knfw and Knrev. For cassette assembly the following reaction mix was used: 200 ng of fragments 91, 92 and *aphA-3*; 20 pM of each primers 91fw and 92rev, 2mM dNTPs, 1xPCR buffer, 4u of Taq polymerase. Amplification was performed by 35 cycles of PCR with following conditions: 94°C – 1 min, 45°C – 1 min, and 72°C – 4 min. Assembled cassette then was cloned into pGEM-T-Easy plasmid (Promega, USA) and the plasmid pGEM-HpyIIIM was formed.

Plasmid Construction pSK-HpylVM (*hp1352*)

Fragments of the *hp1351* and *hp1352* genes of the A45 *H. pylori* strain labeled 1351 and 1352 were amplified using primers gantcfw1, gantcrev1, gantcfw2, gantcrev2 (Supplementary Table 9). Fragment 1351 was digested by restriction endonucleases HindlII and Xhol, and then cloned into the pSK-Kn plasmid, resulting with the plasmid pSK-1351-Kn. Fragment 1352 was digested by restriction endonucleases PstI and Xbal, and then cloned into the pSK-1351-Kn plasmid, resulting in formation of the plasmid pSK-HpyIVM.

Supplementary Table 3. Oligonucleotides used to generate A45 mutant strains *hpy*, *hp91/92* and *hp1352*.

**name**	**sequence 5'–3'**
1Imfw	TACTCGAGTACTGCCCGCTAAAGCC
1IMrev	CCTAAGCTTGCGAGGCAATACGGGGC
2IMfw	CACTGCAGCCACGCACACCACACATCGC
2IMrev	TTTCTAGAGGGTTCTACGGCTGTAGG
X-aph-fw	TCCTCGAGGATCTTTTAGACATCTAAAT
X-aph-rev	CGCTCGAGTCGATACTATGTTATACGCC
91fw	GTCTTAAGACAAGCAATAGG
91revKn	GATGTCTAAAAGATCCGCTCCAACCCCGTTTGACG
92fwKn	GATACAATATGCGGCAGCTGCCGTTATTTGACGC
92rev	AGGATCGCCAATGAGAG
Knfw	GATCTTTTAGACATCTAAAT
Knrev	TCGATACTATGTTATACGCC
gantcfw1	CTCTCGAGGACGCTAATTGCTGATAAATCG
gantcrev1	GAAGCTTTGCGAGACGACCATGACAGCGCC
gantcfw2	CTCTGCAGCCTTGCGCATCTTTTAGTCTTTCG
gantcrev2	ATCTAGAGAAAACTTAAACACTATCATAG

3. Motif enrichment algorithm

The input data required by the algorithm are a list of *k*-mers (by default k = 15) that are likely to bring a modified base in the FASTA format, a list of long *k*-mers (by default k = 29), a reference genome in the FASTA format, signal shifts for each considered short *k*-mer and signal shifts for each long *k*-mer. The optional algorithm parameters are the desired confidence level threshold ***conflevel*** (default is 100 to provide higher sensitivity) and the maximum number of extracted motifs ***max_motifs*** (default is 20).

1) The first stage is the generation of ***motif_variants***, the list of all potential motif variants. An individual motif variant is a vector with size of *k* looking as follows:

$$(b_1, b_2, b_3, \dots , b_{k-2}, b_{k-1}, b_k)$$

where the i-th element represents the nitrogenous base (A, G, T, or C) located in the i-th position of *k*-mer.
The maximum size of ***motif_variants*** is limited by the total set of *k*-mers presented in the reference genome. Since on this step we mainly focus only on R-M systems of types II or III these motif variants should satisfy certain constraints that are common for all Type II-III R-M system recognition sites. These constraints are listed below:
   1. A motif variant has strictly *k* letters.
   2. A motif variant should contain at least 3 non-degenerate letters.
   3. A motif variant should contain either A or C nucleotides (or both).
   4. A motif variant should not contain individual non-degenerate nucleotides surrounded by two N.
   5. A motif variant should not contain more than 8 letters other than N.
   6. A motif variant should not contain more than two sequential N inside.

Constraints 2-6 were inferred using Golden Standard recognition site sequences available in REBASE.
Pattern examples (with *k* = 15):
NGGGCTANNNNNNNN
NNNNGCNNATNNNNN
NNNNNNNNNNCCWGG

2) Next, the algorithm generates the ***reference_set*** list containing all *k*-mers presented in the reference genome. It will be used as a control set. The set of all *k*-mers that are most likely to bring a modified base will be named ***seq_set***.

3) Next, the algorithm performs the chi-square test for each motif presented in ***seq_set*** in order to compare its frequency in ***seq_set*** with the frequency in ***reference_set***. After all motifs in ***seq_set*** are processed, the ***motif_variants*** list is sorted according to the chi-square statistic value in descending order. The top motif is extracted and adjusted.

4) The adjustment process works as follows. All `N` bases in the considered motif variant that are neighboring with canonical bases are iteratively changed to 'A', 'C', 'G', and 'T' and the frequency of resulting motifs is compared with the reference. If the A:C:G:T ratio in one

particular position in ***seq_set*** is close to ***reference_set***, this base remains to be ‘N’. Otherwise, it changed to a more suitable letter (Y, W, S, H, etc). For the adjustment, the algorithm uses only non-filtered ***seq_set*** regardless of iteration.

5) Next, the algorithm checks for the completeness of the extracted motif. It collects all *k*-mers that match the considered motif from the ***reference_seq,*** merges signal levels for these motifs for native and control samples independently and compare them using K-S test. If the K-S test p-value is lower than the threshold, the algorithm considers the motif as incomplete. If the motif is incomplete, the algorithm checks if this motif is a part of a long motif.

   To do that, the algorithm collects all long modified *k*-mers that contain this pattern, and performs a local motif enrichment with the logic similar to the main enrichment algorithms. The algorithm generates a list of all long motif variants, where each long motif variant should satisfy the heuristics generated using the Golden Standard REBASE dataset of long motifs (http://rebase.neb.com/cgi-bin/trdlist?g). For each long motif variant, the algorithm performs the chi-square test, returns 15 motifs with the highest chi-square statistics value and writes to the output file. If the considered short motif is actually a part of a long motif, these 15 long motif variants are quite similar, and the top variant describes the actual long motif sequence. Otherwise, the motif should be considered as short but not fully-methylated in the reference genome.

6) Next, the algorithm checks if the adjusted motif is a submotif for one of the extracted earlier motifs. If it is, the motif is transformed to its supermotif. For example, if we have already extracted the motif ‘NNNCATGNNNN’ with higher confidence level, and the current adjusted motif variant is ‘NNNNCATGWNN’, it will be transformed to ‘NNNNCATGNNN’.

7) Thereafter, the algorithm extracts the adjusted motif variant and performs filtering of the ***seq_set*** by removing all *k*-mers that match this motif variant.

8) If the confidence level of the last extracted motif is less than ***conflevel*** or the number of extracted motifs equals ***max_motifs***, the algorithm stops. Otherwise, the algorithm goes to 3) and repeats processing of the filtered ***seq_set*** to try to extract the next motif.

The algorithm output is the list of extracted motifs.

Long methylation motifs detection example

The main Snapper pipeline is designed to detect only short methylation motifs with a maximum length of 8 bases. However, we developed an additional module that performs local enrichment for motifs that seem incomplete (details are available in Supplementary Materials 3, step 5). As a result, this module proposes a list of longer motif variants and corresponding confidence levels. For example, in *N. gonorrhoeae* FA 1090, the main Snapper pipeline discovers a short GCA motif as methylated but not complete. The long motifs enrichment module supposes that GCA is a TRD1 recognition site sequence and searches for the most probable TRD2 sequence. In this particular case, the best statistics was obtained for GCANNNNNNTGC which is an actual Type I R-M system recognition site in the considered N. gonorrhoeae strain. The algorithm saves the modified contexts containing the target motif so that a user could perform motif enrichment with MEME to additionally approve the motif sequence or ise Nanodisco to define the methylation type.

In cases when TRD2 sequence with satisfactory confidence has not been found, the algorithm concludes that the motif is short but not fully-methylated.

4. J99 extracted motifs

Supplementary Table 4. Snapper output for *H. pylori* J99

MOTIF	conflevel	effsize	comment
NCATGN	30725.1	1.304	
NGCGCN	31789.6	0.85	
NGATCN	28787.6	0.36	extremely high confidence level
NGANTCN	18370.6	0.4	high confidence level
NCCGGN	16504.4	0.98	
NGCCTAN	18232.3	1.32	
NGACAY	12090.2	1.12	these two motifs combining with GTCAC (conflevel = 3847.4) form GWCAY motif
NGTCATN	12902.7	1.33	
NACGTN	10060.5	1.07	
NCCNNGG	8993.5	0.74	
NGAGGN	8984.4	0.28	high confidence level, moderate signal shift
NATTAATN	10201.5	0.95	
NGGWCWAN	10589.9	0.55	These two motifs form GGWCNA motif
NGGWCNAN	9547.2	0.46	
NGTACN	7495.2	1.07	
NCGACGN	6267.5	1.02	these two motifs form CGWCG motif
NCGTCGN	5088.5	0.51	
NTCGAN	4788.8	0.44	Rather low signal shift and confidence level, should be verified manually. 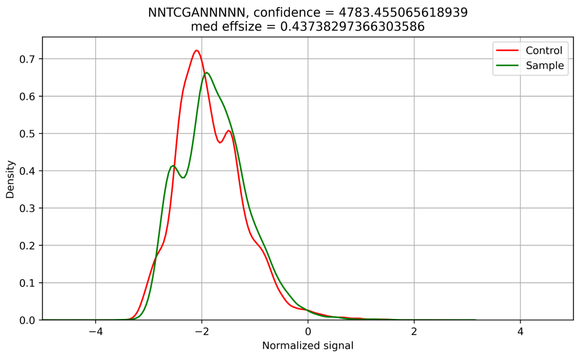

			Actually, the absence of a common mode indicates that it is an individual motif.
NGTCACN	3847.4	1.105	combining with GTGAC (conflevel = 2307.4) forms GTSAC; combining with GACAY and GTCAT forms GWCAY
NGTGACN	2307.4	0.67	combining with GTCAC (conflevel = 3847.4) forms GTSAC
NAAGN	766.1	0.13	was identified as incomplete, automatically extended to AAGNNNNNNCTC (conflevel=193756.5) AAGNNNNNNNTAAAG (conflevel=37314.7)

confidence level legend:
red <= 500
500 < yellow <= 3000
3000 < green

effect size legend:
red <=0.25
0.25 < yellow <= 0.5
0.5 < green

5. TCNNGA motif in J99 strain

Snapper did not extract the TCNNGA motif as methylated while PacBio did. To verify the results we manually observed all the modified context containing this motif. There were 1255 different 11-mers that had a significant signal shift and contained TCNNGA subsequence. Actually, TCNNGA is a quite highly-represented motif in the J99 genome that might cause a false-positive inference. We checked the presence of other motifs in each context and found that almost all of them (1190 contexts) were explained by the presence of one or more other confirmed methylation motifs (**Supplementary Listing 1**). Among the other 65 probably modified contexts more than half (38) contained homopolymeric fragments which as we found might often cause a false-positive signal shift. Generally, despite a quite high number of TCNNGA-containing contexts the number of unexplained contexts containing only TCNNGA but not any other methylation motifs is insufficient for TCNNGA inference. Thus, we can conclude that TCNNGA is not an individual methylation motif in our J99 strain.

Supplementary Listing 1. A shortened list of TCNNGA-contaning contexts that are likely to bring a modified base. The second column shows the presence of a non-TCNNGA motif that explains the signal shift in each case. Only 25 out of 1255 contexts are shown just as an example. In total, 1190 TCNNGA-contaning contexts that have a significant signal change are explained by other motifs.

context	actual motif
TCTCGATCCGC	GATC
GGAATCAAGAA	GANTC
TCAGTCACGAA	GTSAC
TTTTCATGACC	CATG
ATCATGACTGG	CATG
ATCATGAAATT	CATG
TAGAGTCCGGA	CCGG
GCGTGTCCGGA	CCGG
ATCTAGATCCT	GATC
TCTTGATCTAT	GATC
CACTCATGAGA	CATG
TCAAGATCAGA	GATC
TACGATCATGA	CATG
AAAGATCTAGA	GATC
TATCAAGAATC	GANTC
GCTTCATGATC	CATG
TGCTCATGAAT	CATG
ACTCCAGAGGC	GAGG
TGCTCATGATA	CATG
ATCTCCTGATC	GATC
TCAAGATCGCT	GATC
TCTTGATCTTG	GATC
CACTCATGATC	CATG
GATCATGAAAT	CATG
GATCATGATTA	CATG

Supplementary Table 5. The comparison of Snapper, Nanodisco and Tombo on four external datasets.

genome	Motifs detected with Nanodisco (manual mode)	Raw Snapper output	Tombo output	source	comment
Thermacetogenium phaeum DSM 12270 https://www.ncbi.nlm.nih.gov/nuccore/CP003732.1	CGCG	CGCG	CGCG	REBASE & PacBio	
	CCTCC	CCTCC		REBASE & PacBio	
	CAGAAA	CAGAAA		REBASE & PacBio	
	CCCRAG	CCCAAG, CCCGAG		REBASE & PacBio	
	CTACT	CTACT		REBASE & PacBio	
	GATC	GATC	GATC	REBASE & PacBio	
	GGNCC	GGNCC		REBASE & PacBio	
	RAACTC	RAACTC		REBASE & PacBio	
	CCAG	CCAG		this study	these two motif were not mentioned in the reference work1 but were detected by us with both Nanodisco and Snapper
	TGGCCA	TGGCCA		this study	
Neisseria gonorrhoeae FA 1090 https://www.ncbi.nlm.nih.gov/nuccore/NC_002946.2	CCGCGG	CCGCGG		REBASE & PacBio	
	GCCGGC	GCCGGC		REBASE & PacBio	
	GCANNNNNNNNTGC	GCANNNNNNNNTGC		REBASE & PacBio	
	GGCC	GGCC	GGCC	REBASE & PacBio	
	GGNNCC	GGNNCC		REBASE & PacBio	
	GGTGA	GGTGA, TCACC		REBASE & PacBio	complement sequences with non-symmetric modification site
	GTANNNNNCTC	GTANNNNNCTC		REBASE & PacBio	
	RGCGCY	AGCGCC, AGCGCT, GGCGCT		REBASE & PacBio	GGCGCC variant was omitted by Snapper since supermotif GGNNCC had been extracted earlier
	CTNAG	CTNAG		REBASE & PacBio	
	AGCT	AGCT		REBASE & PacBio	

genome	Motifs detected with Nanodisco (manual mode)	Raw Snapper output	Tombo output	source	comment
Methanospirillum hungatei JF-1 https://www.ncbi.nlm.nih.gov/nuccore/NC_007796.1	CCACGK	CCACGK		REBASE & PacBio	
	GATC	GATC	GATC	REBASE & PacBio	
	GCYYGAT	GCCTGAT, GCCCGAT, BGCTCGAT, BGCTTGAC		REBASE & PacBio	here, AGCT was extracted first so BGCTCGAT, BGCTTGAC appeared instead of GCTCGAT and GCTTGAC
	GTAC	GTAC		REBASE & PacBio	
Clostridium perfringens ATCC 13124 https://www.ncbi.nlm.nih.gov/nuccore/NC_008261.1	CCGG	CCGG		REBASE & PacBio	
	CACNNNNNRTAAA	CACNNNNNATAAA*		REBASE & PacBio	*we manually checked the motif correctness here and did not find CACNNNNNGTAAA variants among modified contexts
	GATC	GATC	GATC	REBASE & PacBio	
	GGWCC	GGWCC		REBASE & PacBio	
	GTATAC	GTATAC		REBASE & PacBio	
	VGACAT	VGACAT		REBASE & PacBio	
	WGGCCW	WGGCCW		this study	this motif was detected independently with both Snapper and Nanodisco

6. TGGCCA and CCAG motifs in Thermacetogenium phaeum DSM 12270 (Nanodisco motif refinement)

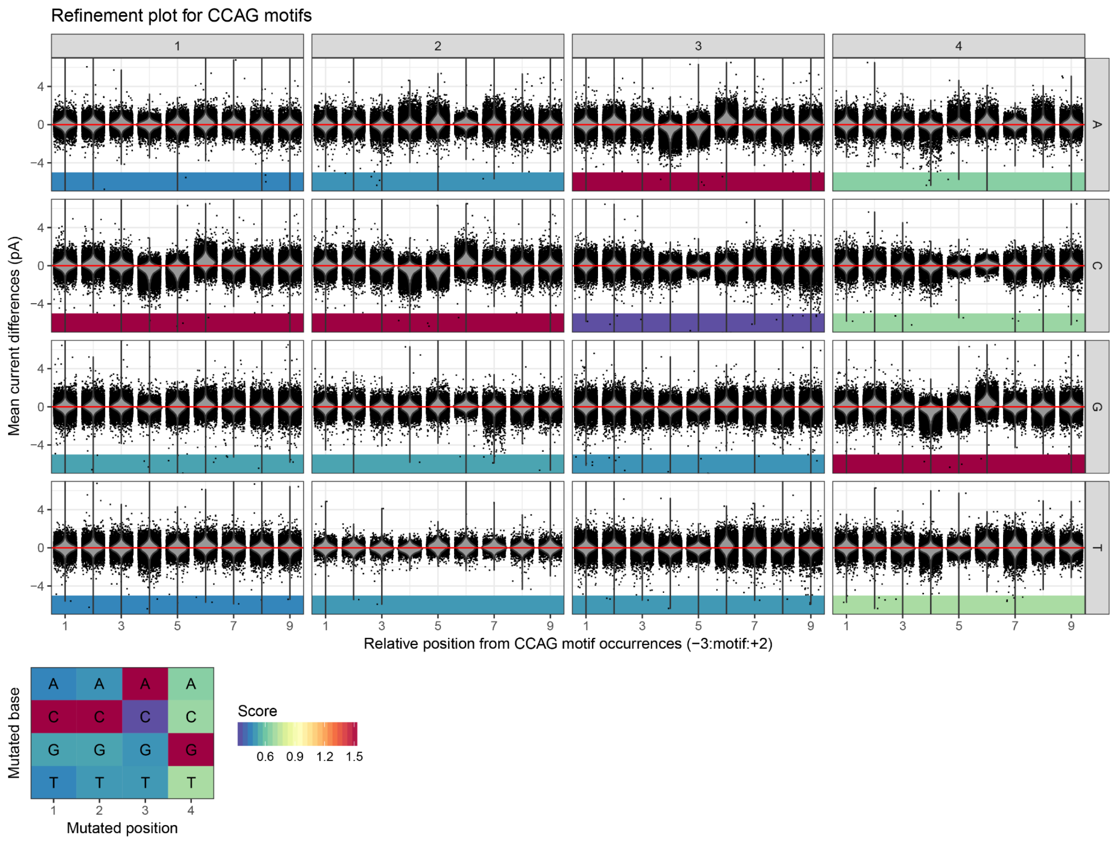

Supplementary Figure 3. Nanodisco refinement plot for CCAG motif detected in *Thermacetogenium phaeum* DSM 12270

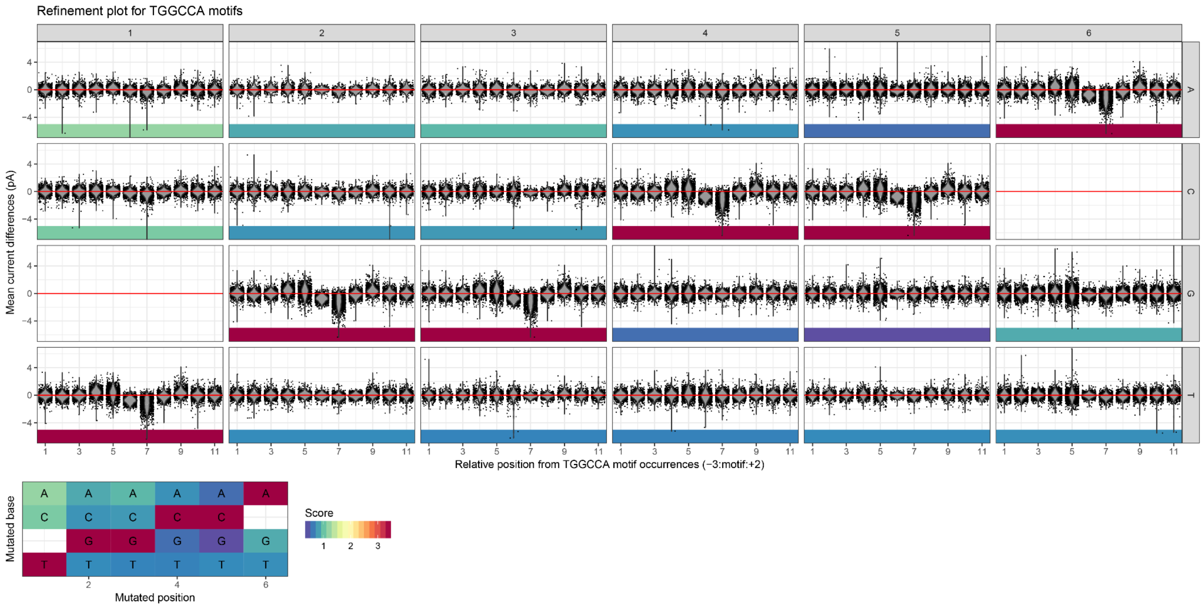

Supplementary Figure 4. Nanodosco refinement plot for TGGCCA motif detected in *Thermacetogenium phaeum* DSM 12270.

7. A45 extracted motifs

Supplementary Table 6. Snapper output for *H. pylori* A45.

MOTIF	conflevel	effsize	comment
NCATGN	32951.2	1.59	
NGCGCN	32109.6	1.11	
NTGCAN	19535.3	1.00	
NGAACN	21296.2	0.79	
NGGCCN	22838.8	1.42	
NGATCN	24323.8	0.46	
NCCAGN	22631.8	0.72	
NCCATCN	29952.0	1.54	
NGAHTCN	25034.2	0.72	should be merged with NGANTC to NGANTCN
NGGGGAN	14548.9	0.58	GGAGA and GGGGA form GGRGA
ATTAATN	16256.6	1.26	
TCNNGAN	16322.6	0.63	
NTCNGAN	18074.7	0.63	
NGANTC	11994.4	0.37	
NGGAGAN	11128.4	0.60	GGAGA and GGGGA form GGRGA
GTNNACN	9591.0	0.92	
NTCGAN	4558.2	0.62	

confidence level legend:
red <= 500
500 < yellow <= 3000
3000 < green

effect size legend:
red <=0.25
0.25 < yellow <= 0.5
0.5 < green

8. *hpy* mutant analysis

Supplementary Table 7. Snapper output for *H. pylori* J99-*hpy* mutant.

MOTIF	conflevel	effsize	comment
NCATGN	70292.8	1.54	
NCCAAKN	68588.4	0.52	

9. *hp1352* mutant analysis

Supplementary Table 8. Snapper output for *H. pylori* J99-*hp1352* mutant.

MOTIF	conflevel	effsize	comment
NGANTCN	99349.1	0.66	

10. *hp91/92* mutant analysis

Supplementary Table 9. Snapper output for *H. pylori* J99-*hp91/92* mutant.

MOTIF	conflevel	effsize	comment
NGATCN	68280.2	0.69	
NCCAATN	63263.7	1.17	submotif for NCCAAK
NCCAAGN	83480.3	0.53	submotif for NCCAAK

11. *hp944* mutant analysis

Supplementary Table 10. Snapper output for *H. pylori* J99-*bc13* mutant.

MOTIF	conflevel	effsize	comment
NCCAGN	83270.4	0.70	

12. Signal distribution shifts in deactivated methylation sites in four A45 mutants.

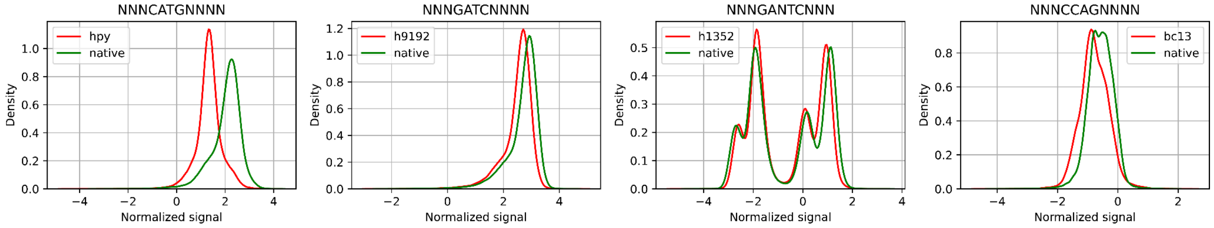

A B C D

Supplementary Figure 5. Signal shifts for inactivated methylation sites in *hpy* (A), *hp91/92* (B), *hp1352* (C) and *hp944* (D) mutants. Here, the green plots represent the motif signal

distribution in the native DNA sample, red plots - the signal distribution in the corresponding mutant.

13. The list of proteins significantly overrepresented in *hpy* and *hp91/92* strains compared with the wild type and the *hp1352* mutant

Non-target proteomic analysis was carried out for wild A45, *hpy*, *hp1352* and *hp91/92*. For each sample, three biological repeats were analyzed, each in 3 technical repeats. In this study the proteomics data were used for identification of a new MTase that turned out to be active only in *hpy* and *hp91/92* mutants but not in the wild type, so, we were interested in the proteins that were significantly overrepresented in *hpy* and *hp91/92* mutants.

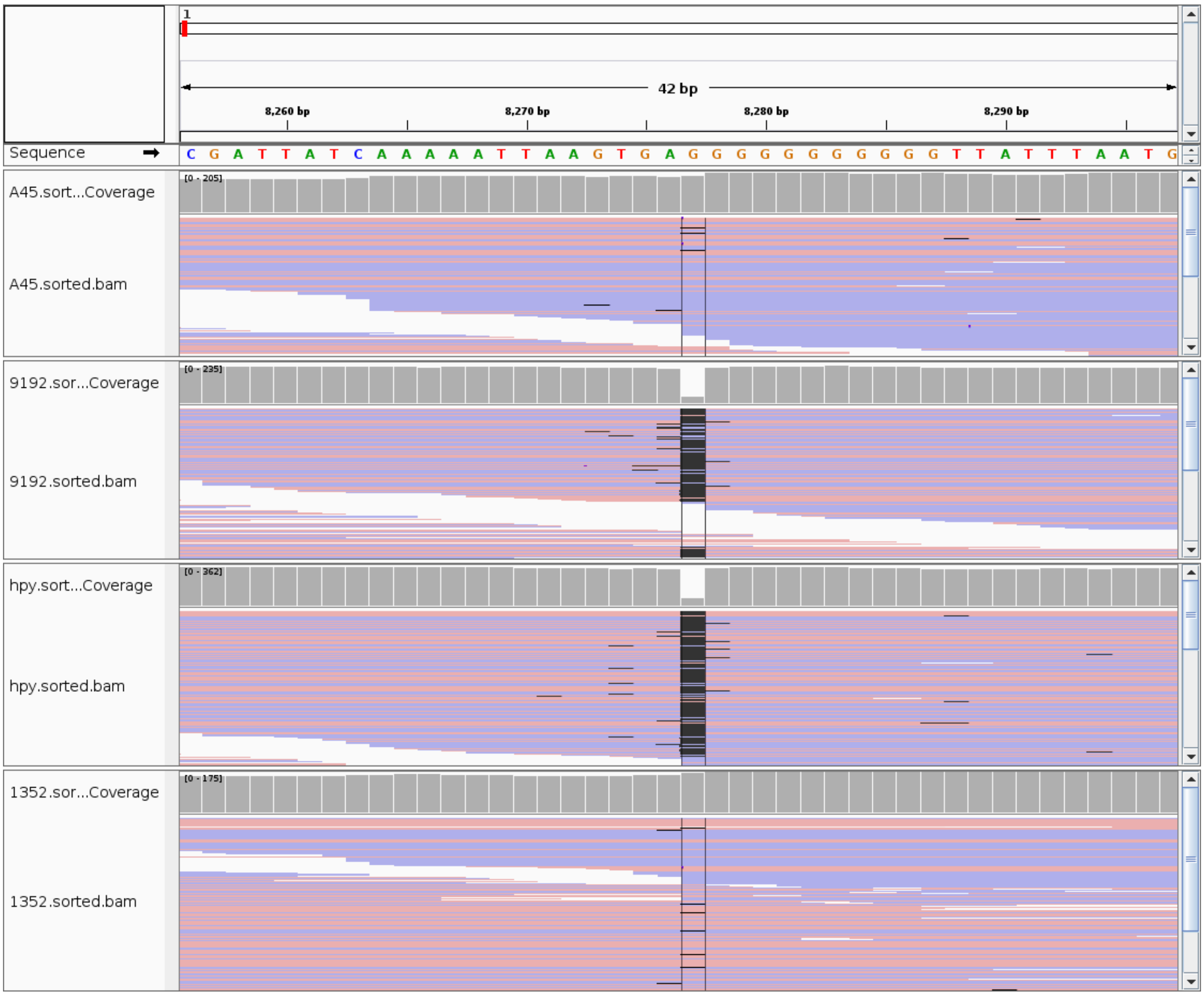

Supplementary Figure 6. Mapping Illumina reads to the reference genome sequence wild strain *H. pylori* A45. IGV snapshots with squished coverage mode, pink and blue segments were aligned to the forward and reverse strand, respectively. A black line represents a deletion with the event size.

14. Proteomics methods

LC-MS analysis

Liquid chromatographic separation was performed on a reverse phase column (15 cm × 75 μm i.d., Agilent Technologies, USA) packed with Zorbax 300SB-C18 resin (particle size – 3 um, pore diameter – 100 A) that was coupled to a Q-Exactive HF hybrid quadrupole-Orbitrap mass spectrometer (Thermo Fisher Scientific, Germany) via a nanoelectrospray source Nanospray Flex (Thermo Fisher Scientific, Germany). Source voltage was set at 2200 V and capillary temperature at 325°C.

Each sample was introduced through EASY-nLC (Thermo, USA) chromatography system in trap-elute configuration (trap column was a 2 cm × 75 μm i.d. Acclaim PepMap column by Dionex, USA, with C18 resin with 3 um-particles with 100 A pores). Samples were introduced onto trap-column with 10 uL of solvent A (0.1% v/v formic acid) at constant pressure 500 bar. Peptides were eluted with a gradient of0 5 to 50 % (v/v) of solvent B (0.1 v/v formic acid, 79.9 v/v acetonitrile) across 60 minutes at flowrate of 500 nl/min in 3 linear steps (10 minutes to 10% B, 25 min to 20% B, 15 min to 35% B, 10 min to 50% B). After each elution system and columns were washed with 100% of solvent B for 10 minutes and regenerated with 5% of solvent B for 20 minutes.

The mass-spectrometer was operated in positive mode in a data-dependent experiment with survey scans acquired at a resolution of 120,000 at m/z 400 within m/z range of 200-1600 with automatic gain control set for 3x106 and maximum injection time of 32 ms. As many as 20 of the most abundant precursor ions with a charge +2 and above from the survey scan were selected for HCD fragmentation. The normalized collision energy was 27. MS2 spectra were acquired at resolution of 7500 at m/z 400, automatic gain control was set for 2x105 and maximum injection time for 32 ms. After fragmentation ions were dynamically excluded from consideration for 45 s.

Quantification

Proteins were relatively quantified using the MaxQuant software version 1.6.10.43. Raw files were searched with an integrated Andromeda search engine against the core peptide database. that consisted of peptides that could be produced by strictly one protein. Trypsin/P was specified as the cleavage enzyme, and two missed cleavages were permissible, minimum peptide length was 7. The FDR was set to 0.01 for peptide spectral matches.

Protein abundance was estimated as a total intensity of its 3 most intense peptides. Difference in each protein's abundance was considered significant if it allowed the FDR to be below 0.05, in addition.

Supplementary Table 11. The list of proteins significantly overrepresented in *hpy* and *hp91/92.*

run ID	Protein IDs					strain
	hp_0008	hp_0009	hp_0010	hp_0927	hp_0928	
LFQ intensity AO0102_1	9810800000	7322500000	10202000000	1190400000	1529000000	*hpy*

LFQ intensity AO0102_2	9364100000	7110000000	10412000000	1596300000	1380800000	*hpy*
LFQ intensity AO0102_3	9217600000	6737600000	10113000000	1658200000	1688300000	*hpy*
LFQ intensity AO0103_1	9362900000	6948900000	9295500000	1165200000	970480000	*hpy*
LFQ intensity AO0103_2	9665300000	6658300000	9460100000	1132600000	998490000	*hpy*
LFQ intensity AO0103_3	8736100000	6376000000	9482000000	1128500000	1245900000	*hpy*
LFQ intensity AO0104_1	9360800000	6591000000	10903000000	1131000000	1323500000	*hpy*
LFQ intensity AO0104_2	8843400000	7027400000	10689000000	1018700000	1583300000	*hpy*
LFQ intensity AO0104_3	9332200000	6635600000	10548000000	1356200000	1537700000	*hpy*
LFQ intensity AO0111_1	5706700000	4080700000	6088600000	375560000	669250000	*hp91/92*
LFQ intensity AO0111_2	5856900000	4130300000	5813000000	475570000	348050000	*hp91/92*
LFQ intensity AO0111_3	5860100000	3564400000	6109200000	538440000	717000000	*hp91/92*
LFQ intensity AO0112_1	10047000000	5009000000	7978300000	645450000	494010000	*hp91/92*
LFQ intensity AO0112_2	5687900000	5605000000	7879400000	425440000	353280000	*hp91/92*
LFQ intensity AO0112_3	6209200000	3999900000	7302800000	416250000	248620000	*hp91/92*
LFQ intensity AO0113_1	6562300000	5895700000	7702500000	201770000	158900000	*hp91/92*
LFQ intensity AO0117_1	0	0	0	0	0	A45
LFQ intensity AO0117_2	0	0	0	0	0	A45
LFQ intensity AO0117_3	0	0	0	0	0	A45
LFQ intensity AO0118_1	0	0	0	0	0	A45
LFQ intensity AO0118_2	0	0	0	0	0	A45
LFQ intensity AO0118_3	0	0	0	0	0	A45
LFQ intensity	0	0	0	0	0	A45

AO0119_1						
LFQ intensity AO0119_2	1145400000	0	0	0	0	A45
LFQ intensity AO0119_3	0	0	0	0	0	A45
LFQ intensity AO0123_1	0	0	0	0	0	*hp1352*
LFQ intensity AO0123_2	0	0	0	0	0	*hp1352*
LFQ intensity AO0124_1	0	0	0	0	0	*hp1352*
LFQ intensity AO0125_1	0	0	0	0	0	*hp1352*
LFQ intensity AO0125_2	0	0	0	0	0	*hp1352*
LFQ intensity AO0125_3	0	0	0	0	0	*hp1352*

Supplementary Table 12. BLAST annotation of the proteins overrepresented in *hpy* and *hp9192* mutants.

gene	protein sequence	annotation
hp0008	MQNKEIGEEKSVKEKNLEVFNRYFPGCLSIENDDKLTLDTG RLKALLGDFSEIKEEGYGLDFVGKKIALNQAFKKNNKILKP LNESTSKHILIKGDNLDALKILKQSYSEKIKMIYIDPPYNTK NDNFIYSDDFSQSNEETLKQLDYSKEKLDYIKNLFGSKCHS GWLSFMYPRLLLAKDLLKQDGVIFISIDDNECAQLKLLCDE IFGEGNFVAEMPRLTKKAGKSTNQIAKNHDYVLCYQKNNI NFKQIDIDENDYPLKDEFYNERGGYKLNQNLDYNSLQYNK KMDYEIVISNEKFYAGGLETYTERQKGNFGTIDWVWRWSK AKFDFGLANGFVEVKNNRIYTKTYTKAKISDSKPYKIEYFN RTKNISSIEFLDNKYSNDMSNKKLQSIFNVKNIFDYSKPVEL ISFLIDQTTEKGDIILDFFAGSGTTAHAVLESNKSDYQKLSEG GGVI	site-specific DNA methyltransferase (broken)
hp0009	MRGGGLFNGLNAAFKERRFILVQLDEKIDPKKNKSAYDFC LNTLKSPSPSIFDITEERIKRAGAKIKEACPHLDVGFRAFEIID DETHANDKNLSQAHQKDLFAYSNPKKRETQTILIKLLGCEG LELTTPINCLIENALYLALNTAFIVGDIEMSEVLENLKDKGV EKISVYMPAISNDRLCLELGSNLLDLKLESGDLKIRG	site-specific DNA methyltransferase (broken)
hp0010	MKIKFKRLDYQEQCRDQILGVFKGIYLREPENDAQRISNPV FEIGEIKDLLLENIENLRSKQKITQGSVGIDKSLNCDILMETG TGKTFCFLECVYSLHKNYHLSKFIVLVPSNAIKLGVLKSVEI TREFFKSEYSTHLESYEDIRSFILASNHKCCVLVMTFSAFNK EKNTINKSCLENTNLFNGAKSYMQALASISPVVIMDEPHRF LGDKTKKYLEQLNALITLRFGATFKDDYKNLIYALDSKKAF	type III restriction-modification system endonuclease

	DCALVKSISVASVGESNECFLELKGVVKIQNGYEAMINYTN LENKIQSVKVKKHDNLGALTQISALEDYIVENITKTEARFLN GFNLLLDQKEPFSHLLEGEQEVMLKEAIKSHFEREEGLFKK GIKALCMVFISGVNSYLSENEKPAKLALLFEKLYQQKLEEV LKKEDLDENYRAYLERTKDNIQKVHGGYFAKSKKESDEAQ VIALILKEKEKLLSFESDLRFIFSQWALQEGWDNPNVMTICK LAPSHSHITKLQQIGRGLRLAVNDKGERITKEHADFDFVNE LVVIVPQVEGDFVGAIQQEISEHSLIKQVFSGEELEKSGIVK KGYYGALLEKLESLGFGEKTDDENFKLTLNQNEFLEKEPEL EKLKDEKYLNLEKLKGFLKDRLIGNSRVRNKNERKSEKIKI NKENFKKFETLWEGLNHQARIAYAIDSESLIDEIVKNIDSSF NVKSKIVSVTTHKKVETMGNNAKTEIFEQKSACVWSLHEFI SALSNKVKLSFKSVAKVLENIDENKFDLIKKNEQESLRRLE ELFLEIIYQNIRDRISYQMRETTIKDRKNDAFYDEKGEIREFL DGSLVGDKYEIKNSSTQEKCLYENFMQVESEIEKDTIEESND TKIIVFGKLPRVKIPIGLNQTYSPDFGYVVENNDKKVLLVVE TKGVENESELHEEEKRKISTAKKFFEALKKQGVNIEYKTKI KKDQLSALINEVLNRKD	
hp0927	MLLEQIQAHSSGFEKFIVKTLGIQNVENFINNWYGKQSLSSF ANNFVPGGLNQALDKIGSSTDAKDLQSFLDKTTFGDILNQ MINQAPLINKLISWLGPQDLSVLVNIALNSITNPSKELTSTISS IGEKALNDLLGEGVVNKIMSNQVLGQMINKIIADKGFGGV YHQGLGSILPKSLQKELEQFGLGSLLGSRGLHNLWQKGNF NFLAKDYVFVNNSSFSNATGGELNFVAGKSIIFNGKNTINFT QYQGKLSFISKDFSNISLDTLNATNGLILNAPRNDISVQKGQ ICVNVLNCMSEKKTNPSTSSAPTDETLEVNANNFAFLGTIK ANGLVDFSKVLQNTTIGTLDLGANATFKANNLIVNNAFNN NSNYRVNISGNLNVVKGAALSTNENGLNVGGDFKSEGSLIF NLNNPTHQTIINVTGASTIMSYNNQTLINLNTQLKQGSYTL MDAKRMLYGYDNQIIRQGSLSDYLKLYTLIDFNGKRMQLN GDSLSYDNQPVNIKDGGLVVSFKDNQGQMVYSSILYDKVQ VSVSDKPMDIHAPSLEYYIQRIQGSGGLNAIKSAGNNSIMW LNELFVAKGGNPLFAPYYLQDNPTEHIVTLMKDITSALGML SNSNLKNNSTDALQLNTYTQQMSRLAKLSNFASFDSTDFSE RLSSLKNQRFADAIPNAMDVILKYSQRDKLKNNLWATGVG GVSFVENGTGTLYGVNVGYDRFIKGVIVGGYAAYGYSGFY ERITNSKSDNVDVGLYARAFIKKSELTFSVNETWGANKTQI NSNDTLLSMINQSYKYSTWTTNAKVNYGYDFMFKNKSIIL KPQIGLRYYYIGMTGLEGVMNNALYNQFKANADPSKKSVL TIDLALENRHYFNTNSYFYAIGGVGRDLLVRSMGDKLVRFI GDNTLSYRKGELYNTFASITTGGEVRLFKSFYANAGVGARF GLDYKMINITGNIGMRLAF	vacuolating cytotoxin domain-containing protein
hp0928	MTYRNSKIDLKNERFSKNRSFKGVKKKIAKKHKAKNLSLI AHAFKTQSNLSASFNKKIFLGLGFVSALSAEDYKSSVYWL NSVNENNSHKSYYVSPLRTWAGGSRSFTQNYNNSQLYIGT KNASATPNNSSIWFGEKGYVGFITGVFKAKDIFITGAVGSG NEWKTGGGAILVFESSNELNANGAYFQNNRAGTQTSWINLI SNNSVNLTNTDFGNQTPNGGFNAMGRKITYNGGIVNGGNF GFDNVDSNGTTTISGVTFNNNGALTYKGGNGIGGSITFTNS NINHYKLNLNANSVTFNNSTLGSMPNGNANTIGNAYILNA NNITFNNLTFNGGWFVFNRPDANVNFQGTTTINNPTSPFVN MTGKVTINPNAIFNIQNYTPSIGSAYTLFSMKNGNITYNNVN	vacuolating cytotoxin domain-containing protein

	NLWNIIRLKNTQATKDNSENATSNNNTHTYYVTYNLGGTL<br>YHFRQIFSPDSIVLQSVYYGANNIYYTNSVNIHDNVFNLKNI<br>NDDRADTIFYLNGLNTWNYTNARFTQTYGGKNSALVFNAT<br>TPWANGAIPKSNSTVRFGGYEGVNWGKTGYITGTFTADRV<br>YITGNMMSGNGAQTGGGATLNFVGATEINIAGADFKNLKT<br>TSQNSYMTFIALGDSSGSGKINVSQSDFYDWTGGGYDFTG<br>NSTFDSVNFNKAYYKFQGAENSYTFKNTNFLAGNFKFQGK<br>TTIEKSVLDDASYSFDGINNAFNEDKFNGGSFNFNAKQVNF<br>SGNSFNGGVFNFNNTPKVSFTDDTFNVNNQFKINGAQTTFT<br>FNKGVIFNMQGLLSSLSVGTTYQLLNAKSVDYKDNNNALY<br>QMLHWISGENPSGKLVDENKTAPSSAKIYNVHFTDNGLTY<br>YIKENFNNGITLTRLCTLGYTHCVNIHNEVFHLKNINNNAS<br>NTVFYLNGMTTWKIAGTGVFTQDYSGANSVLVFNQTTPFL<br>AGANPTSNSVVSFGKTSGAEWGLVGYIQGVFKANQIDITGT<br>IRSGNGAQTGGGATLVFNAEKRLNIANAHLNNDKAGLQDS<br>WMNFIVNNGNLNATNANFSNQTPHGGFNLKANDITFNGGS<br>VSGGGNFGVDNANANGNAVIKNVNFSDNGTLIYKGGENS<br>AGNSLTLENNTFNSYNINARVQNLIFNNNSFNGGSYSFNDT<br>KNTTFKGTNTLINSDPFSRLQGSIAIDNNSIFNIERDLTDNTT<br>YTLLSGNNIKYNNEILADNAFSKNLWNLIHYGGEQGTLLRA<br>DNNTFFVQFTQSNGQKFVFEETFNSSSITYKYFTIHSSLFHT<br>DNDSKDIWSQVRKQFDFIPGKTPVCVGVCYIAPYKNQDLIG<br>SSAFAWSLNFGVTVVGTLLLGSAQEKANDNGGSIWFGKNN<br>LLYLHGNFNATNIFLTNNFNVGNPNAGGGATISFNADETLS<br>ADGLNYTNFQTVAMGLQTSTSQHSWANFNSRLSMEIKNSN<br>FRDFTWGGFNFNSGRIAFENTTFSGWTNINGATESGSSYVN<br>MVANTDLIFTDSILGGGIRYDLKANNIIFNNSQIVIDVSKNV<br>NQSSLNGNVTFNHSRLSVKPNAAINIGDSQTQTTLENASSL<br>SFYNNSVANFNGTTAFNGVSYLNLNPNAQVSFNQANFNNA<br>NVTFYGIPLFGKTPDFGNSARLINFKGNTNFNQATLNLRAK<br>NIHINFQGASTFENNSTMNLAESSQASFNALSVEGETNFNL<br>NNSSLLNFNGNSVFNAPVSFYANNSQISFTKLATFNADASF<br>DLSNNSTLNFQSVLLNGTLNLLGNGANALAINASGNFSFGT<br>QGVLNLSNVNLFDAKNKPLVYNILQAQNIQGLMGNNGYE<br>KIRFYGIQIDKADYSFNNGVHSWSFTNPLNTTETITETLHNN<br>RLKVQISQNGVSNNEMFNLAPSLYFYQKNPYNESSNSYNY<br>TSDKAGTYYLSSNIKSFNQNNKTPGTYNVQNQPLQALHIY<br>NQAITKQDLNMIASLGKEFLPKIANLLSSGALDNLNLNSPN<br>GFETLFGIFEKYGITLNQENWKSLLKIINNFSNTANYDFSQG<br>NLVVGAIKEGQTNTNSVVWFGGEGYKEPCAVGDNTCQMF<br>RQTNLGQLLNSSAPYLGYINANFKAKNIYITGTIGSGNAWG<br>SGGSANVSFESGTNLVLNQANIDAQGTDKIFSYLGQGGIEK<br>LFGEKGLGNALSNIIYEESLNDNAIPKDLANMIPKDLGSKTL<br>SSLLSPTEVNNLLGVSAFKNAIMEILNSKTVGDVFGENGLL<br>NALIL	
